# Phenolic Compounds Reduce the Fat Content in *Caenorhabditis elegans* by Affecting Lipogenesis, Lipolysis, and Different Stress Responses

**DOI:** 10.3390/ph13110355

**Published:** 2020-10-30

**Authors:** Paula Aranaz, David Navarro-Herrera, María Zabala, Ana Romo-Hualde, Miguel López-Yoldi, José Luis Vizmanos, Fermín I. Milagro, Carlos J. González-Navarro

**Affiliations:** 1 Center for Nutrition Research, School of Pharmacy and Nutrition, University of Navarra, 31008 Pamplona, Spain; paranaz@unav.es (P.A.); dnavarrohe@gmail.com (D.N.-H.); mzabalan@unav.es (M.Z.); aromo@unav.es (A.R.-H.); mlyoldi@unav.es (M.L.-Y.); fmilagro@unav.es (F.I.M.); 2 Navarra Institute for Health Research (IdiSNA), 31008 Pamplona, Spain; jlvizmanos@unav.es; 3 Department of Biochemistry and Genetics, School of Sciences, University of Navarra, 31008 Pamplona, Spain; 4 Centro de Investigación Biomédica en Red de la Fisiopatología de la Obesidad y Nutrición (CIBERObn), Instituto de Salud Carlos III, 28029 Madrid, Spain

**Keywords:** obesity, bioactive compounds, resveratrol, apigenin, vanillic acid

## Abstract

Supplementation with bioactive compounds capable of regulating energy homeostasis is a promising strategy to manage obesity. Here, we have screened the ability of different phenolic compounds (myricetin, kaempferol, naringin, hesperidin, apigenin, luteolin, resveratrol, curcumin, and epicatechin) and phenolic acids (*p*-coumaric, ellagic, ferulic, gallic, and vanillic acids) regulating *C. elegans* fat accumulation. Resveratrol exhibited the strongest lipid-reducing activity, which was accompanied by the improvement of lifespan, oxidative stress, and aging, without affecting worm development. Whole-genome expression microarrays demonstrated that resveratrol affected fat mobilization, fatty acid metabolism, and unfolded protein response of the endoplasmic reticulum (UPR^ER^), mimicking the response to calorie restriction. Apigenin induced the oxidative stress response and lipid mobilization, while vanillic acid affected the unfolded-protein response in ER. In summary, our data demonstrates that phenolic compounds exert a lipid-reducing activity in *C. elegans* through different biological processes and signaling pathways, including those related with lipid mobilization and fatty acid metabolism, oxidative stress, aging, and UPR-ER response. These findings open the door to the possibility of combining them in order to achieve complementary activity against obesity-related disorders.

## 1. Introduction

Obesity is characterized by a misbalance between food intake and energy consumption, which leads to an excess of fat accumulation and an increased body weight [1]. The increment in body fat induces oxidative stress and a proinflammatory status, which contribute to premature aging and the development of additional metabolic complications. Therefore, obesity is closely linked to an increased risk for the development of other metabolic syndrome-related diseases, such as type-2 diabetes, cardiovascular disease, or cancer [2,3].

Different strategies have been suggested to reduce the excessive accumulation of fat characteristic of obesity. In this sense, the identification and characterization of bioactive compounds (BACs) capable of regulating energy homeostasis represent a potential tool for the management and prevention of obesity-related diseases [4,5,6]. Among these compounds, polyphenols and phenolic acids have been widely described to exert different beneficial and healthy properties, mainly attributed to their antioxidant and antiaging activities [7,8,9,10]. However, in order to be considered as potential agents against metabolic syndrome, more research is needed to elucidate and characterize the biological processes and mechanisms involved in this regulation.

*Caenorhabditis elegans* (*C. elegans*) has become a popular model for studying the mechanisms of different diseases and physiological processes, including obesity, aging, development, locomotive activity, and neurodegenerative disorders [11,12,13]. In the case of obesity and other metabolic disorders, *C. elegans* has been demonstrated to constitute a powerful model for exploring the genetic basis of fatty acid synthesis and the regulation of fat storage [6]. Due to the highly conserved regulatory pathways of energy homeostasis between mammals and *C. elegans*, this nematode constitutes a powerful in vivo model for the screening of food ingredients and the identification of bioactive molecules with healthy properties, including the ability of modulating fat storage, together with the characterization of the biological mechanism involved in this activity [11,12,14,15,16].

Here, we have investigated the effect of different flavonols (myricetin and kaempferol), flavanones (naringin and hesperidin), flavones (apigenin and luteolin), epicatechin, and other non-flavonoid phenolic compounds (curcumin and resveratrol), as well as phenolic acids (*p*-coumaric, ellagic, ferulic, gallic, and vanillic acids) on *C. elegans* fat accumulation and healthspan. Whole-genome transcriptome analyses of the most effective compounds have revealed different biological mechanisms underlying the fat-reducing activity of these molecules.

## 2. Results and Discussion

In *C. elegans*, more than 400 genes involved in fat storage are evolutionarily conserved with mammals and act in common cellular pathways [14], constituting a convenient in vivo model for the screening of the effects of nutritional perturbations and the fatty acid modulation activity of bioactive components of the diet [11,17]. Although this nematode lacks the adipocytes present in adipose tissue in mammals, *C. elegans* stores fat in lipid droplets in its intestinal and hypodermal cells [13]. These droplets are fat-storing organelles consisting of a hydrophobic core of TAG and cholesterol ester surrounded by a phospholipid monolayer containing several proteins [18]. Due to the transparency of the worm, the fat deposition of these lipid droplets can be easily visualized under microscopy by fat-soluble dyes such as Sudan Black B, Oil red O, and Nile Red [11,14,19]. The quantification of the fluorescent fixative Nile Red lipophilic dye has been demonstrated to be a reliable method to determine the lipid content in different cellular models [20,21,22] and in *C. elegans* [17,23,24,25], and it is a powerful method to determine the fat-reducing activity of different compounds [11,24,25]. Besides, we have previously demonstrated that Nile Red data from L4 significantly correlated with Oil Red data (r = 0.9743, *p* = 0.0048) and total lipid content quantified by TLC (0.9981, *p* = 0.001) [24], showing a highly significant correlation between methods.

In particular, several studies have reported the activity of some flavonoids and phenolic acids that, either supplemented as extracts or as single compounds, prolong *C. elegans* lifespan, [9,10,26,27,28,29,30,31,32,33] and exert antioxidant activities [26,27,29]. However, whether these phenolic compounds can modulate fat storage and energy homeostasis in *C. elegans* is less studied. Besides, differences in the protocols and doses used sometimes make difficult the comparison among studies. The present study provides a screening of the potential lipid-reducing effect of different phenolic compounds in *C. elegans* during larvae development, and clarifies the molecular mechanisms involved for the three with the strongest activity.

### 2.1. Phenolic Compounds Reduce Fat Accumulation in Wild-Type C. elegans

We initially screened the potential role of different phenolic compounds modulating *C. elegans* lipid homeostasis, by determining their effect on lipid accumulation. The mean value, calculated as the fluorescence mean value per pixel, together with the integrated density and the volume of the worms were examined. DMSO-treated worms and Orlistat-treated worms were used as controls [34]. All phenolic compounds (Table 1) and phenolic acids (Table 2) induced a significant reduction of the *C. elegans* lipid content at least at one of the doses tested, with the exception of epicatechin, which promoted an increase in the fat proportion of the worms at the three doses tested. In most cases, this reduction in lipid content was accompanied by a significant decrease in worm volume, as a consequence of the lower fat accumulation in the intestine.

**Figure 1:**
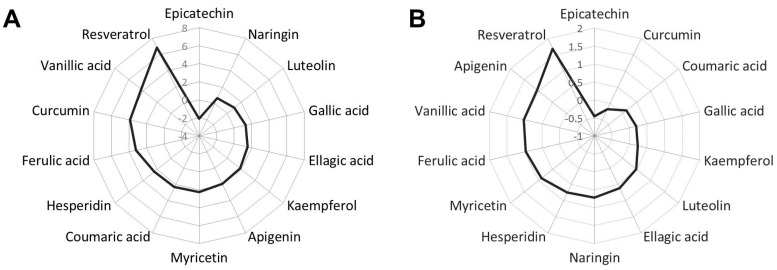
(**A**) Representation of the Cohen’s *d* effect size for the fat-reducing activity of the different phenolic compounds in *C. elegans*, considering Nile Red results from all doses tested. (**B**) Cohen’s *d* effect size of all compounds considering the low (10 µM) and medium (100 µM) doses tested.

In order to determine which compounds induced the higher effects on *C. elegans* lipid content, we calculated the Cohen’s *d* effect size average for each treatment, considering all experiments and doses (10–100–500 µM) evaluated (Figure 1A).

As it can be observed, the phenolic compound with the strongest activity was resveratrol, followed by vanillic acid and curcumin. This is consistent with previous works describing the fat-reducing activity of different phenolic compounds [17,35,36,37,38,39], including phenolic acids [17,23]. In the case of resveratrol, our result would agree with a recent work describing the lipid-reducing activity of trans-trimethoxy resveratrol (TMR), a methyl analog of resveratrol, in this organism [37]. Moreover, it has been previously demonstrated that curcumin-loaded nanoemulsions induced a significant fat reduction in *C. elegans* [39]. As it was mentioned, vanillic was the phenolic acid with the higher activity reducing *C. elegans* fat content, followed by ferulic acid, while gallic, and ellagic acids, which were the least effective. In fact, Saul and colleagues previously observed that treatment with 300 μM of gallic acid and 50 μM of ellagic acid did not significantly reduce the triglyceride content of *C. elegans*, determined photometrically after hydrolysis [9], supporting our results. Finally, Peng and colleagues demonstrated that the treatment with hesperidin on NGM plates containing 10 mM of glucose markedly decreased the fat content of *C. elegans* [36]. Our results demonstrate that this flavonoid can also strongly reduce the nematode fat content in glucose-non-loaded NGM medium.

Due to the fact that the effect size calculated for certain compounds was only attributed to the highest dose used (500 µM), we performed the Cohen’s *d* analysis considering only the low and intermediate doses (10–100 μM) (Figure 1B). In this analysis, we observed that, again, the compound with the strongest activity was resveratrol, followed by vanillic acid and apigenin, so they were selected for further analysis. Thus, we could conclude that, with the exception of epicatechin, treatment with different phenolic compounds during L1 to L4 larvae development significantly reduced the fat content of *C. elegans*.

### 2.2. Resveratrol Treatment during the Larval Stages Improves C. elegans Healthspan

**Figure 2:**
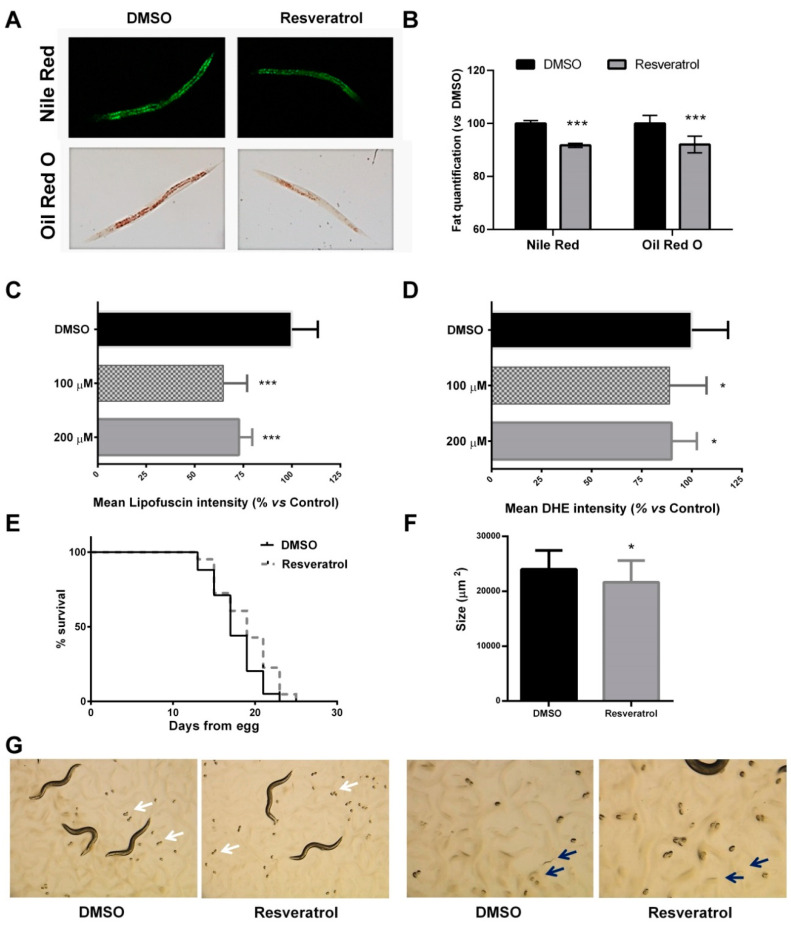
Resveratrol increases *C. elegans* healthspan. (**A**) Nile Red and Oil Red O staining of DMSO- and resveratrol-treated worms. (**B**) Nile Red and Oil Red O quantification of DMSO- and resveratrol-treated worms (100 µM). The results are expressed as the mean ± SD relative to DMSO-treated worms. Significance refers to the effect of resveratrol with respect to DMSO control worms (Student’s *t*-Test, *** *p* < 0.001). (**C**) Quantification of lipofuscin aging pigment of resveratrol-treated worms compared to DMSO control worms (mean ± SD relative to DMSO-treated worms). Significance refers to the effect of resveratrol with respect to DMSO control worms (ANOVA followed by LSD test, *** *p* < 0.001). (**D**) Quantification of the ROS production (measured by DHE) in resveratrol-treated worms compared to DMSO control worms (mean ± SD relative to DMSO treated worms. Significance refers to the effect of resveratrol with respect to DMSO control worms (ANOVA followed by LSD test, * *p* < 0.05). (**E**) Lifespan analysis of resveratrol-treated worms compared to DMSO control worms. (**F**) Size of the treated- and untreated worms on day 1 of adulthood. Significance refers to the effect of resveratrol with respect to DMSO control worms (Student’s *t*-Test, * *p* < 0.05) (**G**) Microscope observation of the presence of eggs (white arrows) and L1 larvae (blue arrows) in both DMSO- and resveratrol-supplemented plates.

As mentioned above, resveratrol exhibited the strongest activity in reducing the fat accumulation of *C. elegans* during its development from L1 larvae to adulthood, and this effect was induced in a dose-dependent manner. The results observed with Nile Red were confirmed by Oil Red O (Figure 2A), showing a significant reduction of the fat content when the nematodes were treated with 100 μM of resveratrol with both staining methods (Figure 2B).

Different studies have demonstrated the anti-obesogenic activities of resveratrol, both in vitro [40,41] and in vivo [42,43,44,45,46]. In *C. elegans*, different works have demonstrated that treatment with resveratrol extended the lifespan of this nematode [47,48,49,50,51,52,53] and reduced oxidative stress [47,51,54]. However, differences in the methodology, selected doses, and length of treatments make the comparison of the results obtained in the different studies difficult.

Thus, we investigated the potential mechanism of the lipid reducing activity of resveratrol by evaluating its effect on *C. elegans* healthspan. *C. elegans* intestinal cells present auto-fluorescence caused by age (sometimes referred to as lipofuscin or age pigment), which is often used as a marker of health or rate of aging [55,56]. Thus, visualization of *C. elegans* fluorescence by microscopy in intact animals revealed that treatment with resveratrol from L1 to L4 caused a significant reduction of the lipofuscin pigment in comparison with the DMSO control worms (Figure 2C). On the other hand, resveratrol treatment induced a significant reduction in the levels of intercellular reactive oxygen species (ROS), determined by dihydroethidium (DHE) [57] (Figure 2D), which suggests the capacity of this stilbenoid alleviating oxidative stress in vivo. Moreover, single treatment with resveratrol (200 μM) from L1 to adulthood was able to induce a significant increase in the worm lifespan, compared with DMSO-treated worms (log-rank Mantel-Cox test, *p* = 0.0019) (Figure 2E). The effect of resveratrol prolonging *C. elegans* lifespan had been previously reported by different authors [47,48,49,50,51,52,53]. However, as far as we know, this is the first time showing that exposure to resveratrol only during larvae development is enough to induce a significant extension in the adult lifespan. Thus, we have demonstrated that the fat-reducing activity of resveratrol is accompanied by an improvement of the *C. elegans* healthspan, demonstrated by the increased lifespan, the reduced aging pigment, and the decreased production of ROS.

Finally, we have also demonstrated that the beneficial effects of resveratrol treatment were independent of effects on the worm development. We observed that worms treated with resveratrol until day 1 of adulthood (day 3 of treatment) showed a reduction in total size, compared with DMSO control nematodes (Figure 2F). However, a qualitative analysis by microscopy demonstrated that both DMSO and resveratrol-treated plates exhibited the presence of eggs (white arrows, Figure 2G) and L1 larvae (blue arrows, Figure 2G), with no differences in the time of appearance of them. This observation indicates that the reduction in the nematode size induced by resveratrol is not explained by an effect on the development, but it is a consequence of the reduction in the content of fat.

In conclusion, these results show that treatment with resveratrol during L1 to adulthood reduces the *C. elegans* fat content with no effects on the worm development, and that this activity is accompanied by a reduction in the cellular aging and the production of ROS, leading to an increased lifespan of the nematode.

### 2.3. Resveratrol Affects Lipogenesis-Related Genes, Stress Resistance, Protein Processing in Endoplasmic Reticulum, and Fatty Acid Metabolism-Related Genes by Mimicking Calorie Restriction Status

The life-expanding activity of resveratrol has been previously attributed to its ability to activate the sirtuin sir-2.1, a member of the Sir-2 family of NAD+-dependent protein deacetylases that regulate nematode aging [50,58,59]. However, other studies have not found this effect of resveratrol on sir-2.1 [52,60,61]. Furthermore, it has not been investigated whether the fat-reducing effect of resveratrol is mediated by this sirtuin.

**Figure 3:**
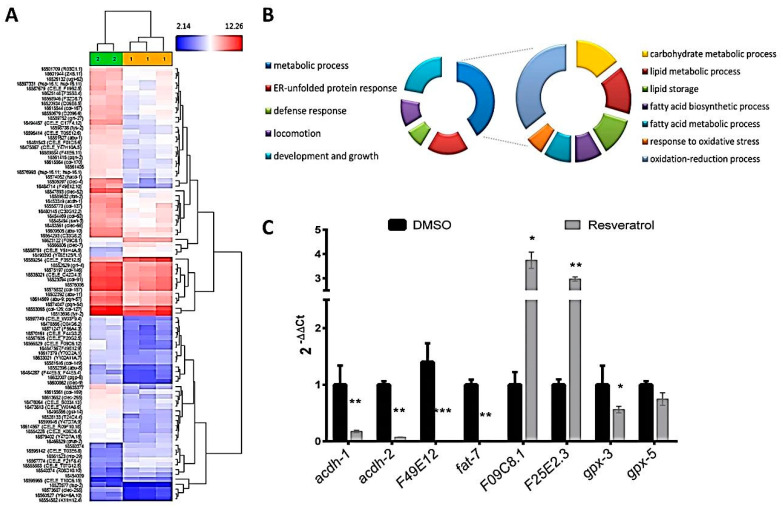
Comparison of the gene expression of resveratrol (200 µM) vs. DMSO-treated worms. (**A**) Hierarchical clustering analysis of control (green) and resveratrol (yellow) samples. (**B**) Gene Ontology biological processes enriched in resveratrol-treated worms compared with DMSO. (**C**) qPCR analysis of genes differentially expressed in microarray analyses. Results are expressed as the fold difference expression levels of each gene in the resveratrol-treated group compared with the DMSO control group, calculated with the 2^−∆∆Ct^ method. Significance refers to the effect of resveratrol with respect to DMSO control worms (Wilcoxon test, * *p* < 0.05; ** *p* < 0.01; *** *p* < 0.001).

For this reason, in order to evaluate the mechanism underlying the fat-reducing and health-promoting activity of resveratrol, we have quantified the transcriptomic response of *C. elegans* to this molecule. Gene-level differential expression analysis, performed by Affymetrix Transcriptome Analysis, revealed a total of 31 genes upregulated and 156 genes downregulated (Table S2) in resveratrol-treated worms (200 µM) compared with DMSO worms (ANOVA *p* value < 0.05; −2 ≥ LogFC ≥ 2; Figure 3A).

The transcriptomic response analyzed by EGAN revealed an enrichment of different Gene Ontology (GO) biological processes and KEGG signaling pathways (Table S3). These results are summarized in Figure 3B, in which we can observe that the fat-reducing activity of resveratrol is mediated by regulation of genes coding for proteins involved in lipid and carbohydrate metabolism processes, such as lipid biosynthesis and fatty acid elongation in mitochondria, fatty acid oxidation, lipid storage, and lipid transport. Thus, genes involved in fatty acid, sterol and other lipid biosynthesis and fatty acid elongation are downregulated after resveratrol treatment compared with DMSO-treated control group (Table 3). By contrast, genes involved in lipid hydrolysis and peroxisomal oxidation are upregulated.

The genes with the strongest downregulation were *F49E12.10* and *F49E12.9* or *drd-1* (dietary restriction down regulated), ortholog of human *FAXDC2* (fatty acid hydroxylase domain containing 2). This protein, highly expressed in liver and adipose tissue, has oxidoreductase activity and is involved in fatty acid and sterol biosynthetic processes. Moreover, resveratrol downregulates some important genes involved in lipid biosynthesis and fatty acid elongation, such as *fat-7*, an ortholog of human Stearoyl-CoA desaturase (*SCD*). This gene codes for an, Δ9 desaturase, a key enzyme in the de novo lipogenic pathway, responsible of the formation of monounsaturated fatty acids from saturated fatty acids by catalyzing the insertion of a double bond to the ninth carbon of saturated C16 and C18 substrates, a needed reaction for the synthesis of triacylglycerol (TAG) and membrane phospholipids and sphingolipids [62]. *Fat-7* and *fat-6* are lipid biosynthesis genes required for lipid droplet expansion, and their expression promotes increased fat stores [63], so downregulation of fat-7 by resveratrol could explain the anti-lipogenic activity of this polyphenol. A similar result was observed by Yue and colleagues after exposure of *C. elegans* to trans-trimethoxy resveratrol (TMR) [37], supporting our observations. Additionally, the inhibitory activity of resveratrol on de novo lipid biosynthesis is also accompanied by the downregulation of other genes involved in fatty acid elongation, such as *acdh-1* and *acdh-2*, orthologs of human *ACADSB* (acyl-CoA dehydrogenase short/branched chain), that encode proteins with medium chain acyl-CoA dehydrogenase activity and oxidoreductase activity, or *cpt-4*, a carnitine palmitoyl transferase ortholog of human *CPT1A*, *CPT1B*, and *CPT1C* genes. Remarkably, *fat-7*, *acdh-1*, *acdh-2*, and *cpt-4* have been previously described as fasting response genes [64,65], as part of the general metabolic response to food deprivation.

Dietary restriction is the most influential environmental intervention to extend lifespan and healthspan in different species, including *C. elegans* [58]. In fact, a previously proposed hypothesis to explain the antioxidant and antiaging activities of resveratrol is that this polyphenol might mimic the molecular response of dietary restriction in different organisms [66]. This hypothesis would be supported by the additional upregulation of genes related to fatty acid hydrolysis and peroxisomal β-oxidation after resveratrol treatment, as *Y51H4A.5*, *F09C8.1* (orthologs of human *PLB1*), and F25E2.3 (ortholog of human *ACOT8)*. PLB1 is a membrane-associated phospholipase that catalyzes the hydrolysis of membrane glycerophospholipids [67]. ACOT8 is a type-2 acyl-CoA thioesterase that catalyzes the hydrolysis of medium- and long-chain acyl-CoAs to free fatty acids and coenzyme A to be utilized for the fatty acid peroxisomal β-oxidation pathway [68]. Thus, the increased expression of these genes by resveratrol demonstrates that, besides the inhibition of de novo lipogenesis, resveratrol activates lipid hydrolysis and peroxisomal β-oxidation, contributing to explain the reduced fat content of the treated worms and supporting the hypothesis that this compound mimics the effect of calorie restriction. Moreover, resveratrol-treated worms exhibited lower expression of different genes involved in response to oxidative stress, such as *F56A4.3* and *gst-14*, orthologs of different human glutathione S-transferases, and the glutathione peroxidase 5 (*gpx-5*), which would demonstrate the activity of this polyphenol reducing the oxidative stress status. All these changes in gene expression were confirmed by qPCR (Figure 3C).

Viswanathan and colleagues have reported that resveratrol induces an overexpression of *abu*/*pqn* (activated in blocked unfolded protein response) genes, involved in the non-canonical unfolded protein response (UPR) [50,69]. In our study, resveratrol-treated worms exhibited an enrichment of various endoplasmic reticulum unfolded protein response (UPR^ER^) GO processes (Table S3). Thus, we observed that this polyphenol induces a general downregulation of different UPR^ER^-related genes (Table 4), including (*abu*/*pqn*) genes, but also the heat-shock protein encoding genes *hps-16* and *hsp-70* (*F44E5.5*), orthologs of human *HSPB2*/*HSPB7*/*CRYAA* and *HSPA14*/*HSPA4*/*HSPH1*, respectively.

Endoplasmic reticulum HSP levels increase in response to enhanced protein damage, as a consequence of exposure to environmental stress conditions, such as presence of oxidants. Besides, these proteins play an important role in aging and longevity [70,71]. Finally, resveratrol also downregulates *aarg-4*, ortholog of the human *GANAB*, that is a key glycoprotein quality control protein in endoplasmic reticulum, where it removes glucose residues from immature glycoproteins, and whose expression has been associated with cellular remodeling during weight regain [72].

The ER is the major subcellular compartment for protein and lipid biosynthesis. Changes in fatty acid desaturation or sterol levels, together with aberrant phospholipid (PL) composition can severely disrupt homeostasis of the endoplasmic reticulum (ER), activating the unfolding-protein response (UPR^ER^) [73,74]. Thus, the general downregulation of all these UPR^ER^-related genes can be explained by the reduced fat synthesis and decreased oxidative stress induced by resveratrol compared with untreated control worms.

In conclusion, the transcriptional response of *C. elegans* exposed to resveratrol is dominated by genes associated with a reduced fat synthesis and an elevated lipid oxidation, which explains the reduced fat content of the treated worms, mimicking the calorie restriction response. As a consequence, endoplasmic reticulum-UPR and glutathione metabolism stress responses are downregulated by this polyphenol, which might be responsible for the antioxidant, antiaging, and the life-expanding effect observed in resveratrol-treated nematodes.

### 2.4. Apigenin Treatment Induces the Oxidative Stress Response and Lipid Mobilization in C. elegans

A recent study has reported the fat-reducing activity of six dietary flavonoids, including apigenin, luteolin, kaempferol, and myricetin in *C. elegans* [35]. In that study, luteolin was the compound with strongest effect on lipid accumulation, being this effect mediated by promoting central serotonin signaling, which in turn induced lipolysis and fatty acid β-oxidation in this nematode [35]. In our study, Nile Red staining indicates that apigenin was the flavonoid with the strongest activity at doses lower than 500 µM, and Oil-Red-O staining confirmed this lipid-reducing activity (Figure S1). For this reason, we aimed to further investigate the underlying mechanism of the effect induced by apigenin, evaluating the transcriptomic response to this polyphenol through whole genome microarrays analyses.

**Figure 4:**
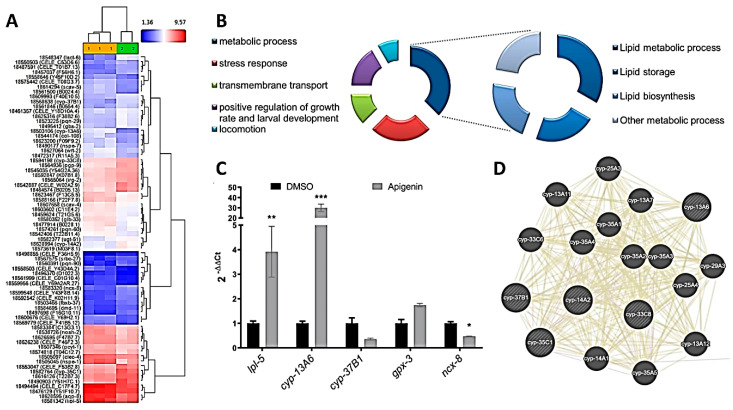
Gene expression microarray comparison of apigenin (100 µM)-treated worms vs. DMSO control worms. (**A**) Hierarchical clustering analysis of apigenin (yellow) and DMSO (green) samples. (**B**) Gene Ontology biological processes enriched in apigenin-treated worms compared with DMSO control group. (**C**) qPCR analysis of genes differentially expressed in microarray analyses. Results are expressed as the fold difference expression levels of each gene in the apigenin-treated group compared with the DMSO control group, calculated with the 2^−∆∆Ct^ method. Significance refers to the effect of resveratrol with respect to DMSO control worms (Wilcoxon test, * *p* < 0.05; ** *p* < 0.01; *** *p* < 0.001). (**D**) Interaction network obtained from GeneMANIA of the most related genes connected with cyp P450 observed after treatment with apigenin.

As it can be observed in Table S4, 34 genes were downregulated and 37 upregulated after apigenin (100 µM) treatment in comparison with DMSO-treated worms (ANOVA *p* value < 0.05; −1.5 ≥ logFC ≥ 1.5; Figure 4A). These genes are involved in different GO processes and KEGG pathways (Table S5), including stress response, defense response, lipid storage, and lipid localization (Figure 4B).

Among them, apigenin induced a strong upregulation of *lipl-5* (Table 5), ortholog for the human genes *LIPA* (lipase A, lysosomal acid type), *LIPF* (lipase F, gastric type), and *LIPM* (lipase family member M), that encodes for a lipase that catabolizes TAG to free fatty acids and glycerol in the response to nutritional and immune stresses [75,76,77]. A previous work observed that *lipl-5* expression is induced by starvation, activating lipid catabolism [75]. Macedo and colleagues also observed that the *lipl-5* gene product participates in lipid homeostasis in both well-fed and starved conditions, with mitochondrial lipids being particularly affected by *lipl-5* mutation [76]. Besides, the expression of this gene has been previously related to reduced lipid droplet size [77], suggesting that the upregulation of this gene by apigenin plays a crucial role in the fat-reducing activity of this flavone.

Moreover, apigenin also significantly downregulates the transport protein-coding genes *ncx-8*, ortholog of the human *SLC8B1* (solute carrier family 8 member B1), which is involved in calcium:sodium antiporter activity and lipid storage [78], and *pgp-9*, ortholog of human *ABCB1* (ATP binding cassette subfamily B member 1). Changes in *lipl-5* and *ncx-8* genes were confirmed by qPCR (Figure 4C).

On the other hand, apigenin significantly affected the gene expression of genes involved in stress response and detoxification, such as *irg-2* (infection response gene), or different genes of the cytochrome P450 family. Thus, apigenin downregulated *cyp-35C1*, *cyp-37b1*, *cyp-14A2*, and *cyp-33C8*, and induced the expression of *cyp-13A6*. The cytochrome (CYP) P450 enzymes (P450s) are phase I detoxification enzymes that catalyze oxidative reactions of a broad spectrum of substrates and play a critical role in the metabolism of xenobiotics, such as drugs and dietary compounds [79], including polyphenols like apigenin [80], whereby a hydroxyl group is introduced to the molecule. Moreover, CYP enzymes have also been found to play a critical function in the synthesis and degradation of lipid steroid hormones, cholesterol, ceramides, polyunsaturated fatty acids (PUFAs), and vitamin A metabolism [79,81]. These CYP proteins are connected with other P450 such as the CYP-35A family members *cyp-35A1*, *cyp-35A2*, *cyp-35A3*, *cyp-35A4*, and *cyp-35A5* (Figure 4D), all of them involved in metabolism of long-chain fatty acids, including linoleic and arachidonic acids [82]. Inactivation of cyp-35 members results in a reduced accumulation of intestinal fat, quantified by Nile Red staining, suggesting that CYPs are involved in lipid storage [82,83]. Besides, the metabolic products of P450 enzymes are more water-soluble and become available to phase II enzymes, such as glutathione peroxidases. In fact, apigenin also induced the upregulation of *gpx-3* (*C11E4*), ortholog of the human glutathione peroxidase 3 (*GPX3*) gene, that encodes for a phase II antioxidant enzyme crucial to maintain redox homeostasis [84]. The upregulation of both *cyp-13A* and *gpx-3* genes, together with the downregulation of *cyp-33C8*, were confirmed by qPCR (Figure 4C).

In summary, treatment of *C. elegans* with the flavonoid apigenin induces a significant reduction in fat deposition, which is mediated by the upregulation of lipid metabolism-related genes, such as *lipl-5*, encoding a triglyceride lipase involved in lipolysis. Moreover, apigenin treatment modulates the expression of different cytochrome P450 monooxygenase genes and the phase II detoxification-encoding gene *gpx-3*, highlighting the importance of these xenobiotic enzymes in the regulation of lipid storage and metabolism.

### 2.5. Vanillic Acid Induces the Oxidative Stress Response and Upregulates Heat Shock Proteins in C. elegans

Different studies have reported the activity of different plant extracts, with variable contents in phenolic acids such as gallic [26,27] and ellagic [23,26], that exert antioxidant activities and prolonged lifespan in *C. elegans* [26,27,85]. Besides, treatment with ferulic and ellagic acids have been previously shown to reduce the *C. elegans* lipid content [17,23]. However, to our knowledge, this is the first time that a report describes the lipid-reducing effect of vanillic acid in this in vivo model. Previously, this phenolic acid has been reported to combat oxidative stress and exert anti-diabetic properties in diabetic hypertensive rats [86]. Thus, as vanillic acid was the phenolic acid with strongest fat-reducing activity in our experiment, we investigated the underlying mechanisms by whole-genome gene expression microarrays.

**Figure 5:**
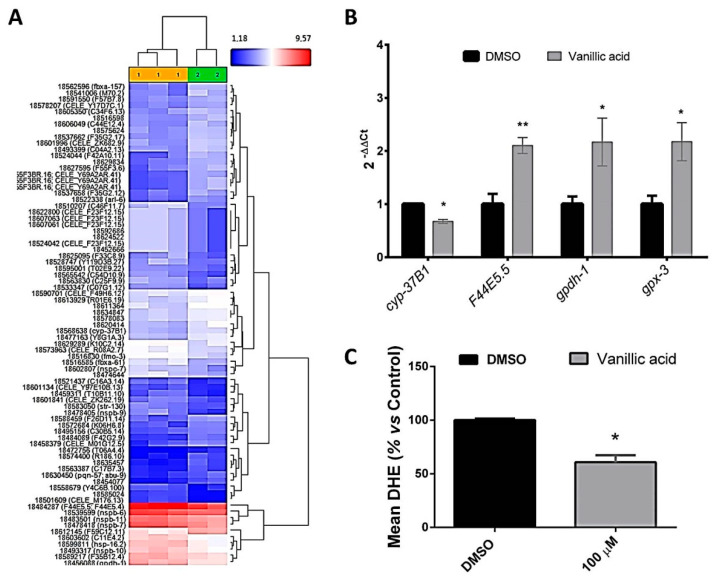
Gene expression microarray comparison of vanillic acid (100 µM)-treated worms vs. DMSO control worms. (**A**) Hierarchical clustering analysis of vanillic acid (yellow) and DMSO (green) samples. (**B**) qPCR analysis of genes differentially expressed in microarray analyses. Results are expressed as the fold-difference expression levels of each gene in the vanillic acid-treated group compared with the DMSO control group, calculated with the 2^−∆∆Ct^ method. Significance refers to the effect of vanillic acid with respect to DMSO control worms (Wilcoxon test, * *p* < 0.05; ** *p* < 0.01). (**C**) Quantification of the ROS production (measured by DHE) in vanillic acid-treated worms compared with DMSO control worms (mean ± SD relative to DMSO-treated worms). Significance refers to the effect of vanillic acid with respect to DMSO control worms (Student’s *t*-Test, * *p* < 0.05).

Gene-level differential expression analysis revealed a total of 58 genes upregulated and 65 downregulated in nematodes treated with vanillic acid (100 µM) in comparison with DMSO control worms (Table S6, Figure 5A).

Surprisingly, in contrast to resveratrol and apigenin, none of these genes corresponded to classical lipid metabolism-related genes and the expression differences were less evident. The main differences in expression were observed in genes involved in endoplasmic reticulum-UPR and other stress responses (Table S7). Thus, and similarly to the result observed with apigenin, vanillic acid induced changes in stress-response genes, such as C11E4.2 or *gpx3*, ortholog of the human glutathione peroxidase 3 gene (*GPX3*) (Table 6), that encodes the antioxidant enzyme that catalyzes the reduction of hydrogen peroxide and lipid hydroperoxides produced during normal metabolism [87]. GPX-3 deficiency has been associated with cardiovascular disease and stroke [88].

Moreover, vanillic acid induced the downregulation of some cytochrome P450-related genes, such as *cyp-37b1*, ortholog of human cytochrome P450 family 4 members *CYP4A11*, *CYP4B1*, and *CYP4F3*, which encodes an oxidoreductase expressed in the worm intestinal cells. This gene has been found to be modulated in response to food deprivation, and its activity has been related to endogenous fatty acid metabolism and fat mobilization in *C. elegans* [65]. The expression changes in the antioxidant *gpx-3* and *cyp-37B1* genes by vanillic acid were confirmed by qPCR (Figure 5B) and might be explain the antioxidant and the lipid-reducing activity of this phenolic compound. Moreover, as shown in Figure 5C, vanillic acid-treated worms exhibited a reduced ROS accumulation (quantified by DHE) in comparison with the control group (Student’s *t*-Test, *p* = 0.034), confirming the in vivo antioxidant capacity of this compound in *C. elegans*.

On the other hand, vanillic acid significantly upregulated *gpdh-1* (Figure 5B), which is expressed in *C. elegans* hypodermis and intestine. *Gpdh-1* corresponds to the ortholog of the human Glycerol-3-Phosphate dehydrogenase (*GPD1*) gene and GPD1L (glycerol-3-phosphate dehydrogenase 1 like). GPD1 is member of the NAD-dependent glycerol-3-phosphate dehydrogenase family which catalyzes the reversible redox reaction of dihydroxyacetone phosphate (DHAP) and NADH to glycerol-3-phosphate (G3P) and NAD+ [89]. Thus, the encoded protein catalyzes the rate-limiting step of glycerol biosynthesis and plays a critical role in carbohydrate and lipid metabolism. Inactivating mutations in this gene have been found to be the cause of different cases of transient infantile hypertriglyceridemia [90] and fatty liver disease [91]. Moreover, GPD1L has been found to be upregulated during weight loss and weight maintenance induced by low calorie diet (LCD), but downregulated during weight gain induced by high-fat diet (HFD) [92]. In *C.elegans*, deletion of *gpdh-1* results in decreased stress resistance, so its upregulation by vanillic acid might be related with the antioxidant capacity of this phenolic compound, which might also underly the fat-reducing activity observed by Nile Red and Oil Red O staining methods.

Finally, as it was mentioned before, a significant activation of the unfolded protein response of the endoplasmic reticulum (UPR^ER^) was observed after vanillic acid treatment, as it can be observed by the significant upregulation of the genes encoding for the heat-shock proteins *hsp-16.2*, *hsp-70* (or *F44E5.5*) and *hsp-16.48*, orthologs of human *HSPB1*, *HSPA8*, and *HSPB2*, respectively (Table 5) [70]. The upregulation of *hsp-70* (*F44E5.5*) by vanillic acid was confirmed by qPCR (Figure 5B). The UPR^ER^ is induced by proteotoxic conditions, but also by a lipid disequilibrium. Endoplasmic reticulum, as a membrane-bound organelle, is sensible to changes in lipid desaturation and metabolism [93]. In *C. elegans*, it has been demonstrated that a compromised fatty acid desaturation and a reduced phosphatidylcholine production are able to activate the UPR^ER^ without inducing proteostatic imbalance [74]. Thus, the increased UPR^ER^ observed after vanillic acid treatment might be a consequence of the reduced fat content induced by this phenolic compound.

**Figure 6:**
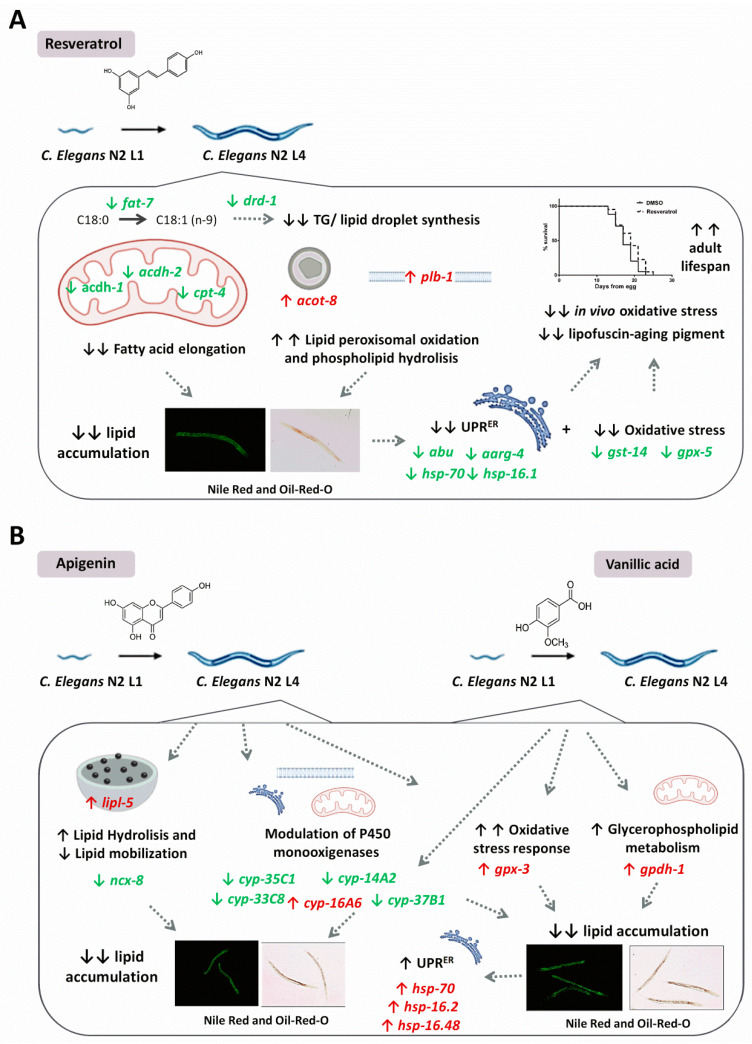
(**A**) *C. elegans* treatment with resveratrol during the L1 to L4 stages mimics the calorie restriction response, as suggested by the reduced expression of the lipid synthesis-related genes *fat-7* and *drd-1*, together with a reduced lipid mobilization and elevated peroxisome lipid oxidation and hydrolysis, which explains the reduced fat content of the treated worms. As a result, endoplasmic reticulum-UPR and glutathione metabolism stress responses are downregulated, which might be responsible for the improvement of *C. elegans* health and lifespan by this phenolic compound. (**B**) *C. elegans* treatment with apigenin induced a significant upregulation of the triglyceride lipase gene *lipl-5*, together with changes in the expression of cytochrome P450 monooxygenase genes and *gpx-3*, which might explain the reduced lipid content of the worms treated with this compound. Similarly to apigenin, vanillic acid treatment also affected the expression of the antioxidant genes *cyp-37B1* and *gpx-3*, suggesting an increased response to oxidative stress in the treated worms. Besides, vanillic acid induced the expression of the glycerophospholipid metabolism-related gene *gpdh-1*, which might be partially responsible of the fat-reducing activity of this compound in *C. elegans* and the subsequent activation of the ER-UPR.

In conclusion, our data demonstrates that vanillic acid treatment induces small changes in *C. elegans* gene expression, mainly affecting to genes involved in the antioxidant and stress resistance response, which might underlie the fat-reducing activity observed by this phenolic compound. Besides, the changes in the lipid content after treatment induced the activation of the UPR^ER^, highlighting the importance of this organelle in the regulation of lipid synthesis and metabolism. Although further research in needed to investigate the effect of vanillic acid on mammalian metabolism, our data suggest that this compound, together with the phenolic compounds resveratrol and apigenin, could be useful in the prevention of the excessive fat accumulation characteristic of obesity. Besides, the different mechanisms involved in the fat-reducing activity of each phenolic compound (Figure 6) suggest the possibility of a future evaluation of their combination as a potential therapeutic agent in obesity-related diseases.

## 3. Materials and Methods

### 3.1. Reagents

Chemicals were obtained from SIGMA Aldrich (St. Louis, MO, USA): Hesperidin analytical standard, ≥97.0% (HPLC, ref. # 50162; PubChem CID: 10621); Naringin ≥95% (HPLC, ref. #71162; PubChem CID: 442428); Apigenin ≥97% (TLC) from parsley powder (ref. #A3145; PubChem CID: 5280443); Luteolin analytical standard, ≥97.0% (HPLC, ref. # 72511; PubChem CID: 5280445); Kaempferol ≥90% (HPLC, ref. # K0133; PubChem CID: 5280863); Myricetin analytical standard, ≥98% (HPLC, ref. #72576; PubChem CID: 5281672); Resveratrol ≥99% (HPLC, ref #R5010; PubChem CID: 445154); Curcumin from *Curcuma longa* (Turmeric) powder (ref. #C1386; PubChem CID: 969516); p-Coumaric acid ≥98.0% (HPLC, ref. #C9008; PubChem CID: 637542); Ellagic acid powder from tree bark ≥95% (HPLC, ref. #E2250; PubChem CID: 5281855); trans-Ferulic acid >99%, (ref. #128708; PubChem CID: 445858); Gallic acid 97.5-102.5% (titration), (ref. #G7384; PubChem CID: 370); Vanillic acid purum ≥97.0% (HPLC, ref. #94770; PubChem CID: 8468). All reagents were dissolved in dimethyl sulfoxide (DMSO).

### 3.2. C. elegans Culture and Phenolic Compounds Treatment

*C. elegans* was cultured as previously described [24,94]. N2 Bristol strain, which was obtained from the *Caenorhabditis Genetics Center* (CGC, University of Minnesota, MN, USA) was grown at 20 °C on Nematode Growth Medium (NGM) with *Escherichia coli* OP50 as normal nematode diet.

All assays were performed in triplicate in 6-well cell culture plates with 4 mL of NGM per well. Orlistat supplemented plates (6 µg/mL Orlistat; Sigma-Aldrich, St. Louis, MO, USA) were used as fat reduction control [24,34]. Supplemented media was prepared as described [95] with some modifications. All phenolic compounds were dissolved in dimethyl sulfoxide (DMSO) and tested at concentrations from 10 to 500 µM. The same amount of DMSO (0.1%) was added to non-supplemented plates as control. Once compounds were added to NGM, plates were left to solidify and dry in a dark environment to protect them from light oxidation. Afterwards, 200 µL of an overnight culture of *E. coli* OP50 was seeded and plates were incubated in darkness at room temperature until dry [24]. 

For all experiments, age-synchronized L4 worms were obtained by standard hypochlorite treatment of gravid animals. The eggs were allowed to hatch overnight in M9 medium and about 750 L1 larvae per well were transferred onto plates and grown during 46 h until L4 stage, in which worms were collected and experiments were performed.

### 3.3. Nile Red Staining

Nile Red (#N3013, Sigma-Aldrich, St. Louis, MO, USA), a dye for neutral lipids, staining was performed as previously described [24,25,96] with minor modifications. Briefly, L4 worms were harvested in 1.5 mL tubes and washed two times with PBST (0.01% of Triton X-100 in Phosphate Buffered Saline). Then, worms were kept on ice for 15 min to stop pharyngeal activity and fixed in 40% isopropanol for 3 min. The staining was performed adding 150 µL of Nile Red solution (3 µg/mL) to fixed worms followed by incubation (30 min) at 20 °C in the dark with gentle rocking. Finally, worms were washed in PBST and mounted on a 2% agarose pad for microscopy visualization.

### 3.4. Oil Red O Staining

ORO staining was performed as described [16,24]. The day previous to the staining of the worms, the fresh ORO solution was prepared by diluting stock (0.5% ORO in isopropanol) to a 60% solution with water, filtering (0.45 µM filter), stirring at room temperature overnight and filtering again just before use. At that moment, L4 worms were collected, washed, and fixed in 60% isopropanol for 5 min. Then, fixed worms were incubated in the ORO solution for 6 h in a wet chamber with gentle shaking in the dark, washed with PBS and mounted on a 2% agarose pad for microscopy visualization.

### 3.5. DHE Staining

ROS levels in vivo were measured using the fluorescent dye dihydroethidium (DHE; Dihydroethidium BioReagent, ≥95% (HPCE), Sigma-Aldrich, St. Louis, MO, USA), as described previously [54,97] with some modifications. Briefly, synchronized 500 L1 larvae were transferred onto plates containing DMSO (control) or resveratrol (200 µM), and were grown until the L4 stage. At that moment, L4 worms were collected, washed three times in PBS and maintained in a 3 µM DHE solution (in PBS) during 30 min. Afterwards, worms were washed and mounted on 2% agarose pads with a 1% of sodium azide.

### 3.6. C. elegans Aging Visualization

Synchronized 500 L1 larvae were transferred onto plates containing DMSO (control) or resveratrol (200 µM) and were grown until the L4 stage. Worms were collected, washed, and mounted onto 2% agarose pads with a 1% of sodium azide. Lipofuscin pigment was determined as marker of aging by autofluorescence [98].

### 3.7. Image Acquisition and Quantification

For all conditions tested, approximately 300 animals were fixed and stained. Fluorescent images of Nile Red stained worms were captured at 100× magnification on a Nikon SMZ18 research stereomicroscope equipped with an epi-fluorescence system and a DS-FI1C refrigerated color digital camera (Nikon Instruments Inc., Tokyo, Japan). Images were taken at the same conditions and integration time under a GFP filter (Ex 480–500; DM 505; BA 535–550). For the ORO analysis, images were also captured at 100× magnification with a Nikon SMZ18 research stereomicroscope equipped with a Nikon DS-Fi2 high-definition color camera. The dihydroethidium (DHE)-labeled ROS formation and the lipofuscin autofluorescence were detected by measuring the fluorescence intensity using a Nikon Eclipse 80i epi-fluorescent microscope, equipped with a TRITC filter (Ex 540–625; DM 565; BA 605–655) and the DAPI filter (with excitation at 340–380 nm and emission at 435–485 nm), respectively (Nikon Instruments Inc., Tokyo, Japan). In all cases, the image analysis of the Nile Red, ORO, DHE, and lipofuscin assays was performed using ImageJ software as previously described [24]. The mean value, calculated as the fluorescence mean value per pixel, together with the integrated density and the volume of the worms were determined. Approximately 25–40 worms were examined in three independent experiments for each condition.

### 3.8. Lifespan Analysis

*C. elegans* lifespan analyses were performed in the same manner for all treatments at 20 °C. Synchronized L1 larvae were transferred to NGM plates containing DMSO (control group) or resveratrol 200 µM for 46 h, to allow *C. elegans* to develop to L4 stage. Four replicates were used per condition. At least 50–65 L4 larvae per replicate were transferred then onto new plates containing 40 µM of 5-fluoro-2-deoxyuridine (FUDR, #856657, Sigma-Aldrich, St. Louis, MO, USA), with no additional treatments. Surviving or dead animals were counted daily, until all nematodes died. Worms were scored as dead when they failed to respond to a gentle touch with a platinum wire.

### 3.9. Egg Lying and Worm Size

Egg lying was observed in young adult nematodes (day 3 of growth) grown on NGM agar plates supplemented or not with the polyphenol. The images were taken at 135× magnification using a Nikon SMZ18 stereomicroscope equipped with a Nikon DS-Fi1C high-definition color camera. Worm size (µM^2^) was calculated with the Nikon NIS-ELEMENTS Software (Nikon Instruments Inc., Tokyo, Japan).

### 3.10. Cohen’s d Effect Size

In order to compare the effect of the different compounds and doses used in reducing the *C. elegans* fat content (quantified by Nile Red), the Cohen’s *d* effect size [24,99,100] was calculated using the formula [99]

*d* = (M1 − M2)/σ_p_,

where σ_p_ corresponds to the pooled standard deviation:

σ_p_ = √(σ12 + σ22)/2

### 3.11. RNA Extraction and Quantitative PCR Analysis

Total RNA from *C. elegans* N2 strain was extracted using Trizol^®^ RNA isolation reagent (Thermo Fisher Scientific, Paisley, UK) according to the manufacturer’s instructions. Concentration and purity of RNA were determined at 260/280 nm using a NanoDrop ND-1000 spectrophotometer (Thermo Fisher Scientific, Wilmington, DE, USA). Then, 1000 ng of RNA was treated with DNAse (Ambion™ DNase I, RNase-free; Thermo Fisher Scientific Inc., Waltham, MA, USA) according to the manufacturer’s protocol. For the quantitative gene expression analyses, DNA-free RNA was reverse-transcribed into cDNA following the protocol previously described [24,25].

Gene expression analyses were performed by quantitative real-time PCR (qPCR) using the TaqMan Universal PCR master mix and specific probes (Table S1) from Applied Biosystems Technologies (Thermo Fisher Scientific Inc., Waltham, MA, USA) and Integrated DNA Technologies Inc. (Coralville, IA, USA). All reactions were performed using a CFX384 Touch™ Real-Time PCR Detection System (BioRad, Hercules, CA, USA). The expression level of each gene was normalized comparing to the expression of the *pmp3* and *tba1* genes from Life Technologies (TaqMan Gene Expression Assays), which were used as housekeeping gene controls [101]. Gene expression differences between treated and untreated worms were quantified using the relative quantification 2^−∆∆Ct^ method [102].

### 3.12. Whole-Genome Transcriptomic Analysis

Microarray hybridization was performed by the Genomics Unit of the Center for Applied Medical Research (CIMA, University of Navarra, Pamplona, Spain). Three microarrays were used for each condition. Initially, the quality of total RNA from N2 *C. elegans* was evaluated using the Experion™ Automated Electrophoresis System (BioRad Laboratories, Inc., Hercules, CA, USA). RNAs were then hibridizated in Affymetrix GeneChip^®^ *C. elegans* Gene 1.0 ST arrays using the Affymetrix technology (Thermo Fisher Scientific-Affymetrix, Santa Clara, CA, USA). Background correction and normalization were performed by algorithm Robust Multiarray Analysis (RMA) and then evaluated using the Transcriptome Analysis Console (TAC) v3. (Affymetrix). Probesets were filtered by ANOVA *p* value (*p* < 0.05) and fold change (−2.0 ≤ FC ≥ 2.0). Then, the biological interpretation of the experiment was performed using the Exploratory Gene Association Networks (EGAN) program, from UCSF (University of California at San Francisco, CA, USA). Biological effect categories were selected by Gene Ontology Process and KEGG tools. Gene-gene functional interaction networks were performed with *GeneMANIA* [103].

### 3.13. Statistical Analyses

*C. elegans* body fat reduction (Nile Red and Oil Red O) between treatments and control condition (NGM group), together with oxidative stress (DHE) and lipofuscin determinations, were evaluated by a hierarchical ANOVA test, where replicates were nested in treatments, followed by multiple comparison (Fisher’s protected Least Significant Difference, LSD) tests. For lifespan assays, log-rank (Mantel-Cox test) between resveratrol and control (NGM) treatments were performed. Real-time PCR data was analysed using Wilcoxon test comparing each treatment to its control. All tests were performed using StataSE v12 software (StataCorp LLC, College Station, TX, USA).

## 4. Conclusions

In conclusion, our study describes the lipid-reducing activity of different phenolic compounds and phenolic acids in *C. elegans* during larvae development, with resveratrol, apigenin, and vanillic acid being the most effective ones. Whole-genome expression analyses demonstrate that this activity is mediated by changes in the expression of genes involved in lipid synthesis, oxidation, and mobilization, but also involves other crucial biological processes, such as the cellular response to oxidative stress and UPR^ER^, evidencing the importance of these processes in metabolism. Although further research would be necessary using mammalian obesity models, our data supports the potential application of phenolic compounds, including phenolic acids, as bioactive compounds in the prevention of the excessive accumulation of fat characteristic of metabolic diseases. Furthermore, given the different mechanism of action, our results would open the possibility of new studies in which the synergy between these phenolic compounds could be verified, which may be relevant for the maintenance of the energy homeostasis in obesity-related diseases.

## Figures and Tables

**Table 1:** *C. elegans* lipid quantification data (relative to NGM) obtained after phenolic compounds treatment and measured by Nile Red staining. Data correspond to the mean ± SEM. *, **, *** show the level of statistical significance of the differences, *p* < 0.05, *p* < 0.01, and *p* < 0.001, respectively.

Treatment	Mean Fat Content	Integrated Density	Volume	Sample Size (*n*)
NGM	100.0 ± 0.7	100.0 ± 1.3	100.0 ± 1.3	490
Orlistat	76.3 ± 0.9 ***	54.5 ± 1.4 ***	59.4 ± 1.9 ***	246
Hesperidin				
10 µM	95.0 ± 1.0 ***	90.8 ± 1.6 ***	94.8 ± 1.5 **	201
100 µm	94.7 ± 0.9 ***	87.4 ± 1.4 ***	90.2 ± 1.4 ***	204
500 µM	92.6 ± 1.5 ***	87.3 ± 1.8 ***	90.9 ± 1.2 ***	105
Naringin				
10 µM	101.3 ± 1.6	105.6 ± 2.4 *	108.2 ± 1.8 **	118
100 µM	95.1 ± 1.1 ***	96.9 ± 2.5	102.3 ± 3.9	146
500 µM	99.4 ± 2.4	99.4 ± 3.2	99.2 ± 3.2	98
Curcumin				
10 µM	91.6 ± 1.3 ***	79.5 ± 2.4 ***	81.9 ± 2.5 ***	115
100 µM	101.5 ± 1.6	86.3 ± 3.0 ***	82.5 ± 2.9 ***	124
500 µM	85.8 ± 0.8 ***	53.7 ± 0.9 ***	51.9 ± 1.1 ***	110
Resveratrol				
10 µM	101.7 ± 1.1	105.9 ± 1.7 **	103.9 ± 1.4	125
100 µM	88.1 ± 0.5 ***	77.0 ± 0.9 ***	82.0 ± 1.1 ***	211
500 µM	68.2 ± 1.0 ***	56.4 ± 1.9 ***	63.6 ± 1.0 ***	190
Myricetin				
10 µM	96.5 ± 0.8 ***	91.7 ± 1.4 ***	92.9 ± 1.5 ***	167
100 µM	94.2 ± 0.7 ***	85.3 ± 1.3 ***	86.7 ± 1.1 ***	207
500 µM	95.4 ± 0.8 ***	89.7 ± 1.3 ***	91.8 ± 1.2 ***	100
Kaempferol				
10 µM	96.3 ± 0.7 ***	91.0 ± 1.3 ***	92.1 ± 1.3 ***	157
100 µM	98.4 ± 0.8	91.3 ± 1.5 ***	90.4 ± 1.3 ***	166
500 µM	94.1 ± 0.8 ***	86.1 ± 1.3 ***	88.7 ± 1.3 ***	125
Epicatechin				
10 µM	105.3 ± 1.6 ***	111.8 ± 2.7 ***	109.6 ± 2.4 ***	110
100 µM	103.5 ± 0.9 **	103.2 ± 1.7	99.3 ± 1.5	150
500 µM	107.1 ± 1.3 ***	110.4 ± 2.3 ***	101.5 ± 1.8	104
Apigenin				
10 µM	98.30 ± 0.78	97.82 ± 1.34	98.60 ± 1.15	183
100 µM	93.39 ± 0.84 ***	89.52 ± 1.55 ***	92.83 ± 1.56 ***	218
500 µM	95.96 ± 0.87 ***	88.74 ± 1.52 ***	89.33 ± 1.30 ***	130
Luteolin				
10 µM	101.0 ± 0.8	103.9 ± 1.6 *	104.1 ± 1.4 *	172
100 µM	97.0 ± 0.8 ***	96.6 ± 1.4 *	99.0 ± 1.4	210
500 µM	96.0 ± 0.8 ***	92.6 ± 1.4 ***	95.6 ± 1.3 *	126

**Table 2:** *C. elegans* lipid quantification data (relative to NGM) obtained after phenolic acids treatment and measured by Nile Red staining. Data correspond to the mean ± SEM. *, **, *** show the level of statistical significance of the differences, *p* < 0.05, *p* < 0.01, and *p* < 0.001, respectively.

Treatment	Mean Fat Content	Integrated Density	Volume	Sample Size (*n*)
NGM	100.0 ± 0.9	100.0 ± 1.6	100.0 ± 1.4	353
Orlistat	75.3 ± 1.1 ***	62.3 ± 1.8 ***	72.0 ± 2.2 ***	169
*p*-Coumaric acid				
10 µM	97.8 ± 0.9	103.0 ± 2.0	108.7 ± 2.3 **	124
100 µM	97.0 ± 0.9	97.0 ± 1.7	99.7 ± 1.8	180
500 µM	91.9 ± 0.9 ***	94.6 ± 1.8 **	103.4 ± 2.2	92
Ellagic acid				
10 µM	97.7 ± 1.2 *	102.0 ± 2.0	108.8 ± 2.5 **	133
100 µM	93.6 ± 1.0 **	94.6 ± 1.9	100.5 ± 2.2	174
500 µM	96.8 ± 1.1	96.3 ± 1.9	98.8 ± 2.1	103
Ferulic acid				
10 µM	94.3 ± 0.9 ***	95.0 ± 2.0	101.8 ± 2.2	182
100 µM	92.1 ± 1.0 ***	88.7 ± 2.1 ***	95.3 ± 2.3	190
500 µM	91.3 ± 0.9 ***	91.4 ± 1.7 **	100.3 ± 1.7	119
Gallic acid				
10 µM	94.2 ± 0.9 ***	90.8 ± 1.2 ***	93.5 ± 0.9 ***	142
100 µM	96.0 ± 1.4 **	94.9 ± 2.0 **	96.0 ± 1.6 *	136
500 µM	96.5 ± 1.1 **	92.9 ± 1.6 ***	94.2 ± 1.2 ***	120
Vanillic acid				
10 µM	94.8 ± 1.1 ***	92.9 ± 1.9 ***	97.4 ± 1.6	126
100 µM	89.2 ± 1.1 ***	86.3 ± 1.7 ***	94.5 ± 1.4 *	187
500 µM	86.6 ± 0.8 ***	80.6 ± 1.4 ***	89.3 ± 1.3 ***	134

**Table 3:** Most affected genes involved in lipid and carbohydrate metabolism after treatment with resveratrol.

Gene	Description	Human Ortholog	logFC	ANOVA *p* Value
**Fatty acid and sterol biosynthesis**				
*F49E12.10*	Sterol biosynthesis	*FAXDC2* (fatty acid hydroxylase domain containing 2)	−13.27	0.0007
*F49E12.9/drd-1*	Dietary restriction downregulated-1	*FAXDC2* (fatty acid hydroxylase domain containing 2)	−4.16	0.0054
**Lipid biosynthesis and fatty acid elongation in mitochondria**				
*acdh-1*	Oxidoreductase activity	*ACADSB* (acyl-CoA dehydrogenase short/branched chain)	−3.90	0.0010
*W03F9.4*	Carnitine *O*-palmitoyltransferase activity		−3.64	0.0035
*C06E8.5*	Lipid binding activity	*BPIFA2* (BPI fold containing family A member 2)	−3.21	0.0249
*hacd-1*	3-hydroxyacyl-CoA dehydrogenase activity and NAD+ binding activity	*HADH* (Hydroxy-Acyl-CoA Dehydrogenase)	−2.90	0.0276
*cpt-4*	Carnitine palmitoyl transferase	*CPT1A*, *CPT1B*, and *CPT1C*	−2.27	0.0098
*W02F12.2*	*N*-acylsphingosine amidohydrolase activity. Sphingosine and ceramide biosinthesis	*ACER1* (alkaline ceramidase 1)	−2.22	0.0465
*fat-7*	Fatty acid and lipid biosynthesis. stearoyl-CoA 9-desaturase activity	*SCD* (stearoyl-CoA desaturase) and *SCD5*	−2.15	0.0210
*C06G1.1*	Lipid binding activity	*BPIFA3* (BPI fold containing family A member 3)	−2.14	0.0147
*acdh-2*	Oxidoreductase activity	*ACADSB* (acyl-CoA dehydrogenase short/branched chain)	−2.08	0.0304
**Fatty acid oxidation and hydrolysis**				
*F09C8.1*	phospholipase activity	*PLB1* (phospholipase B1)	3.16	0.0050
Y51H4A.5	hydrolase activity	ND	3.01	0.0064
*F25E2.3*	acyl-CoA hydrolase activity	*ACOT8* (acyl-CoA thioesterase 8)	2.16	0.0111
**Fatty acid and gluthathione metabolism**				
*F56A4.3*	glutathione transferase activity	*GSTA5* (glutathione S-transferase alpha 5); *GSTM1* (glutathione S-transferase mu 1); and GSTM3 (glutathione S-transferase mu 3)	−2.66	0.0397
*gst-14*	glutathione transferase activity	*GSTA4* (glutathione S-transferase alpha 4), *GSTA5* (glutathione S-transferase alpha 5), and HPGDS (hematopoietic prostaglandin D synthase)	−2.53	0.0154
*tyr-2*	Oxidoreductase activity	*DCT* (dopachrome tautomerase)	−2.44	0.0280
*mlt-7*	peroxidase activity	*EPX* (eosinophil peroxidase)	−2.33	0.0447
*C11E4.1/gpx-5*	glutathione peroxidase activity	*GPX5* (glutathione peroxidase 5) and GPX6 (glutathione peroxidase 6)	−2.10	0.0186

**Table 4:** Most affected genes involved in UPR^ER^ after treatment with resveratrol.

Gene	Description	Human Ortholog	logFC	ANOVA *p* Value
*abu-10*	Activated in Blocked Unfolded protein response	ND *	−3.06	0.0017
*F44E5.5*	Heat shock protein 70	*HSPA14* (heat shock protein family A (Hsp70) member 14) *HSPA4* (heat shock protein family A (Hsp70) member 4) *HSPH1* (heat shock protein family H (Hsp110) member 1)	−3.00	0.0367
*abu-1*	Activated in Blocked Unfolded protein response	ND	−2.99	0.0231
*abu-9*	Activated in Blocked Unfolded protein response	ND	−2.94	0.0121
*hsp-16.1*	Heat Shock Protein	*HSPB2* (heat shock protein family B (small) member 2) *HSPB7* (heat shock protein family B (small) member 7) *CRYAA* (crystallin alpha A)	−2.92	0.0404
*abu-8*	Activated in Blocked Unfolded protein response	ND	−2.91	0.0301
*F32D8.7*		*AMBP* (alpha-1-microglobulin/bikunin precursor) *LRP11* (LDL receptor related protein 11) *SPINT1* (serine peptidase inhibitor, Kunitz type 1)	−2.87	0.0479
*D2096.6*		ND	−2.67	0.0229
*F41E6.11*		ND	−2.58	0.0120
*hsp-16.11*	Heat Shock Protein	*HSPB2* (heat shock protein family B (small) member 2) *HSPB7* (heat shock protein family B (small) member 7) *CRYAA* (crystallin alpha A)	−2.45	0.0296
*abu-11*	Activated in Blocked Unfolded protein response	ND	−2.45	0.0135
*CELE_T06E4.8*		ND	-2.27	0.0126
*abu-6*	Activated in Blocked Unfolded protein response	ND	−2.22	0.0192
*abu-5*	Activated in Blocked Unfolded protein response	*KRTAP10-1* (keratin associated protein 10-1) *KRTAP10-4* (keratin associated protein 10-4) *KRTAP10-7* (keratin associated protein 10-7)	−2.18	0.0204
*pqn-74*		ND	−2.03	0.0282
*aagr-4*	carbohydrate binding activity and hydrolase activity, hydrolyzing *O*-glycosyl compounds	*GANAB* (glucosidase II alpha subunit)	−2.01	0.0493

* ND: Not Defined.

**Table 5:** Main genes affected after treatment with apigenin (100 µM) compared with DMSO control worms.

Gene	Description	Human Ortholog	logFC	ANOVA *p* Value
**Lipid metabolism and transmembrane transport**				
*lipl-5*	Lipoprotein lipase-5. Lipid storage, degradation, lipid homeostasis and localization. TAG catabolism	*LIPA* (lipase A, lysosomal acid type); *LIPF* (lipase F, gastric type) *LIPM* (lipase family member M)	1.73	0.0431
*pgp-9*	ATP-binding cassette (ABC) transporter superfamily. Transmembrane transport	*ABCB1* (ATP binding cassette subfamily B member 1) *ABCB11* (ATP binding cassette subfamily B member 11)	−1.77	0.0037
*ncx-8*	Na/Ca exchangers. Lipid storage Lipid storage, localization, and transmembrane transport.	*SLC8B1* (solute carrier family 8 member B1)	−1.67	0.0097
*pcyt-1*	Phosphocholine cytidylyltransferase. choline-phosphate. Phosphatidylcholine biosynthetic process, lipid metabolic process, phospholipid biosynthetic process	*PCYT1B* (phosphate cytidylyltransferase 1, choline, beta).	1.5	0.0070
*gba-2*	Beta-Glucocere Brosidase with hydrolase activity. Lipid metabolic process, sphingolipid metabolic process and carbohydrate metabolic process	*GBA* (glucosylceramidase beta)	−1.53	0.0487
**Stress response and detoxification**				
*cyp-35C1*	Cytochrome P450 family with oxidation-reduction function, xenobiotic metabolic process	*CYP2E1* (cytochrome P450 family 2 subfamily E member 1) *CYP2C18* (cytochrome P450 family 2 subfamily C member 18) *CYP2D7* (cytochrome P450 family 2 subfamily D member 7 (gene/pseudogene))	−1.94	0.0194
*cyp-37B1*	Cytochrome P450 family with oxidation-reduction function	*CYP4A11* (cytochrome P450 family 4 subfamily A member 11) *CYP4B1* (cytochrome P450 family 4 subfamily B member 1) *CYP4F3* (cytochrome P450 family 4 subfamily F member 3)	−1.65	0.0124
*irg-2*	Infection response gene. Innate immune response	ND *	−1.62	0.0229
*cyp-14A2*	Cytochrome P450 family with oxidation-reduction function. Monooxygenase activity	*CYP2U1* (cytochrome P450 family 2 subfamily U member 1)	−1.61	0.0252
*cyp-33C8*	Cytochrome P450 family with oxidation-reduction function. Fatty acid metabolism	*CYP2J2* (cytochrome P450 family 2 subfamily J member 2)	−1.56	0.0003
*gpx-3* (C11E4.2)	Glutathione peroxidase activity Glutathione metabolism Arachidonic acid metabolism Response to oxidative stress Stress response	*GPX3* (glutathione peroxidase 3)	1.73	0.0010
*cyp-13A6*	Cytochrome P450 family. Stress response. monooxygenase activity involved in oxidation-reduction process. Xenobiotic response	*CYP3A4* (cytochrome P450 family 3 subfamily A member 4)	2.81	0.0005

* ND: Not Defined.

**Table 6:** Top upregulated and downregulated genes based on fold change (with ANOVA *p* < 0.05) following vanillic acid treatment (100 µM).

Gene	Function	Human Ortholog	logFC	ANOVA *p*-Value
**UPR^ER^ response genes**				
*pqn-57*	Prion-like-(Q/N-rich)-domain-bearing protein. Involved in endoplasmic reticulum unfolded protein response.	ND *	−1.78	0.0317
*hsp-16.48*	Unfolded protein binding activity. IRE1-mediated unfolded protein response	*HSPB1* (heat shock protein family B (small) member 1), *HSPB2* (heat shock protein family B (small) member 2) and *CRYAA* (crystallin alpha A)	1.51	0.0110
*hsp-16.2*	Unfolded protein binding activity	*HSPB1* (heat shock protein family B (small) member 1), *CRYAA* (crystallin alpha A), *CRYAB* (crystallin alpha B)	1.98	0.0111
*F44E5.5*	Unfolded protein binding activity, misfolded protein binding activity, ATP binding activity	*HSPA14* (heat shock protein family A (Hsp70) member 14); *HSPA4* (heat shock protein family A (Hsp70) member 4); and *HSPH1* (heat shock protein family H (Hsp110) member 1	2.04	0.0341
**Other stress-response genes**				
*C11E4.2/gpx-3*	Glutathione peroxidase activity	*GPX3* (glutathione peroxidase 3)	1.84	0.0027
*cyp-37B1*	Cytochrome P450 family member with oxidoreductase activity involved in defense response to Gram-positive bacterium.	*CYP4A11* (cytochrome P450 family 4 subfamily A member 11); *CYP4B1* (cytochrome P450 family 4 subfamily B member 1); and *CYP4F3* (cytochrome P450 family 4 subfamily F member 3).	−1.63	0.0172
*fmo-3*	Monooxygenase activity	*FMO5* (flavin containing dimethylaniline monoxygenase 5)	1.71	0.0099
*irg-5/CELE_F35E12.5*	Defense response to Gram-positive bacterium.	ND	1.53	0.0393
**Other genes**				
*gpdh-1*	glycerol-3-phosphate dehydrogenase (quinone) activity and glycerol-3-phosphate dehydrogenase [NAD+] activity	*GPD1* (glycerol-3-phosphate dehydrogenase 1) and *GPD1L* (glycerol-3-phosphate dehydrogenase 1 like)	1.7	0.0130
*arl-6*	Exhibits GTPase activity. Is involved in intracellular transport	*ARL6* (ADP ribosylation factor like GTPase 6)	−1.75	0.0267
*dop-3*	Exhibits dopamine neurotransmitter receptor activity, coupled via Gi/Go	*DRD2* (dopamine receptor D2)	−1.53	0.0205
*aat-7*	l-amino acid transmembrane transporter activity.	*SLC7A11* (solute carrier family 7 member 11); *SLC7A7* (solute carrier family 7 member 7); and *SLC7A9* (solute carrier family 7 member 9)	1.52	0.0027
*ugt-63*	UDP-glycosyltransferase activity	ND	1.52	0.0076
*glb-33*	Heme binding activity and oxygen binding activity	ND	1.54	0.0222
*F35B12.4/piit-1*	Serine-type endopeptidase inhibitor activity	*TFPI2* (tissue factor pathway inhibitor 2)	1.63	0.0420
*str-130*	G protein-coupled olfactory receptor activity	ND	1.7	0.0226

* ND: Not Defined.

## Supplementary Materials

The following are available online at https://www.mdpi.com/1424-8247/13/11/355/s1; Figure S1: (**A**) Nile Red and Oil Red O staining of DMSO, orlistat (6 µg/mL, used as positive control), apigenin, and vanillic acid (100 µM)-treated worms. (**B**) Nile Red quantifications of DMSO and treated worms. (**C**) Oil Red O quantifications of DMSO and treated worms. The results are expressed as the mean ± SD relative to DMSO-treated worms. Significance refers to the effect of the different treatments in comparison with DMSO control worms (ANOVA followed by LSD test, *** *p* < 0.001), Table S1: Gene expression probes used for Qpcr, Table S2: Differentially expressed genes after treatment with resveratrol (200 µM) vs. DMSO control worms (ANOVA *p* < 0.05; −2 ≤ FC ≥ 2). Results obtained in Transcriptomic Array Console (TAC, Affymetrix), Table S3: Main GO processes and KEGG signaling pathways affected by treatment with resveratrol (200 µM). Analysis obtained from EGAN UCSF software, Table S4: Differentially expressed genes after apigenin (100 µM) treatment vs DMSO control worms (ANOVA *p* < 0.05; −1.5 ≤ FC ≥ 1.5). Results obtained in Transcriptomic Array Console (TAC, Affymetrix), Table S5: Main GO processes and KEGG signaling pathways affected by treatment with apigenin (100 µM). Analysis obtained from EGAN UCSF software, Table S6: Differentially expressed genes after treatment with vanillic acid (100 µM) vs DMSO control worms (ANOVA *p* < 0.05; −1.5 ≤ FC ≥ 1.5). Results obtained in Transcriptomic Array Console (TAC, Affymetrix), Table S7: Main GO processes and KEGG signaling pathways affected by treatment with vanillic acid (100 µM). Analysis obtained from EGAN UCSF software.
