# Supplementary material for: Phenolic Compounds Reduce the Fat Content in Caenorhabditis elegans by Affecting Lipogenesis, Lipolysis, and Different Stress Responses
Source: Pharmaceuticals (Basel). 2020 Oct 30;13(11):355. doi: 10.3390/ph13110355 (PMC7693530; doi:10.3390/ph13110355)
Supplement: Supplementary file 1 [file pharmaceuticals-13-00355-s001.pdf]

Supporting figures

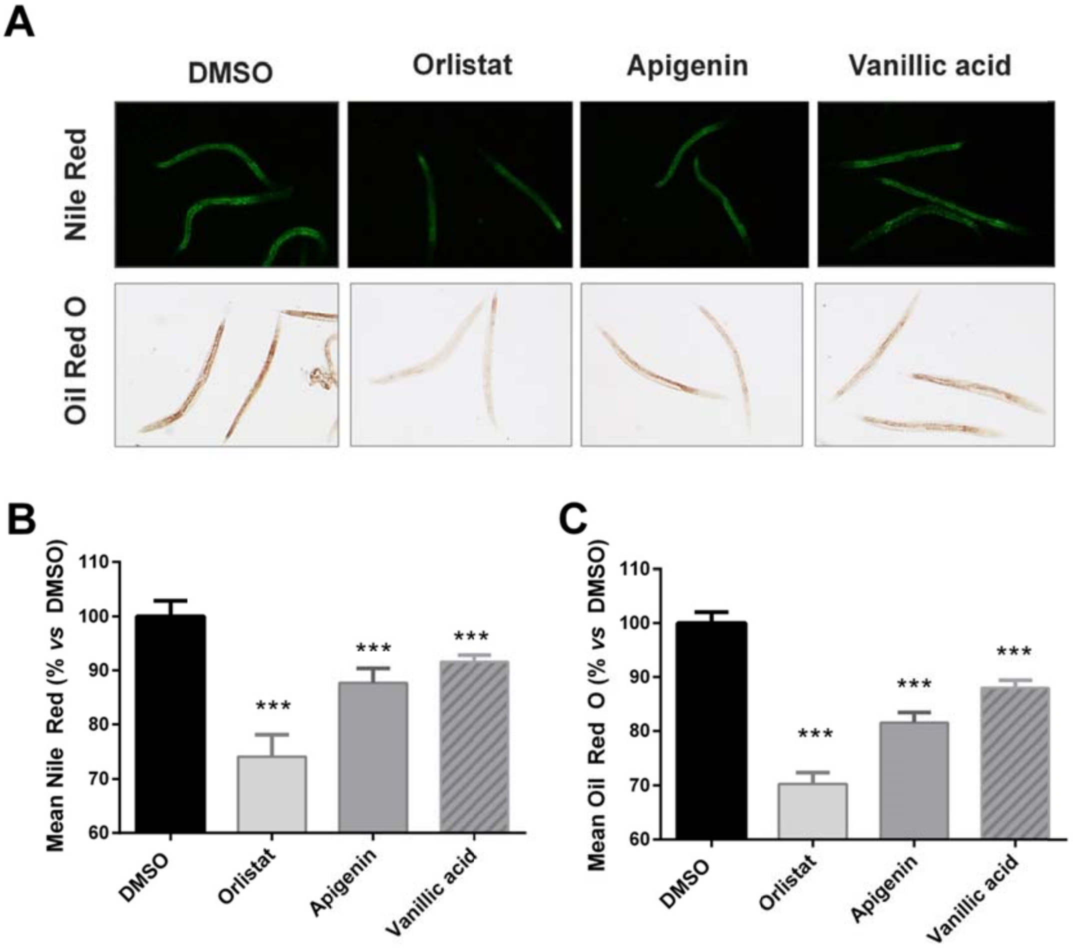

Figure S1. A) Nile Red and Oil Red O staining of DMSO, orlistat (6µg/mL, used as positive control), apigenin and vanillic acid (100 µM)-treated worms. B) Nile Red quantifications of DMSO and treated worms. C) Oil Red O quantifications of DMSO and treated worms. The results are expressed as the mean ± SD relative to DMSO-treated worms. Significance refers to the effect of the different treatments in comparison with DMSO-control worms (ANOVA followed by LSD test, *** $p < 0.001$).

Supplementary Tables

Table S1. Gene expression probes used for qPCR *.

C.elegans gene	Fw sequence	Rv sequence	Probe sequence
fat-7	ACAAGTTAAGGAGCATGGAGG	TCTCCTTCCAGAAATAAACGGG	/56-FAM/CAGAGAAAG/Zen/CACTATTTCCCACTGGTCA/3IABkFQ/
gpx-3	TATGAACGGGCTTACCTATGTG	CAACAGTTTGTGGGCAAGAC	/56-FAM/CTCGTAAAG/Zen/TGGGTGATGATTGTCTCCG/3IABkFQ/
F49E12.10	TTTGTATGGCTATGGATGAAATTGG	TGCGTGTTCAAGTGGATGAG	/56-FAM/ATGCTGGAA/Zen/ATCGAAACTGGTGCG/3IABkFQ/
acdh-1	ATCCGAGCTTCATCCACTTG	CAGTCCAATCATTTGTGCTCC	/56-FAM/AGCCTTTTC/Zen/CATACTCTCCCAGAATTGC/3IABkFQ/
F25E2.3	AGATACTGAGCCAATGTCGATG	CCGTTTCTGATATGAGGTCGTAC	/56-FAM/ATCACCGTC/Zen/TCCATCAGTGCGTC/3IABkFQ/
F56A4.3	AAGAGATTGTGCAGACTGGAG	CAGAGCTTCTATCAGGCATCC	/56-FAM/TGCGTCATT/Zen/TGGGAAGAGTTCATGGA/3IABkFQ/
F09C8.1	TTCTAATGTCTTGTCACCTCCG	TCAAGCTGATCATGGCACTAG	/56-FAM/CGAGGATCC/Zen/GGTAGCAGTCGTTCT/3IABkFQ/
acdh-2	TGAGCTAACCCGACCATTTG	CAGGAACCATTGCTCAAGTG	/56-FAM/ACGCTATTG/Zen/AAGTTCTGAATGCCAGTCG/3IABkFQ/
c17c3.1	AAGAGCATCGAAGAGCAGAAG	TTTTGGATGAGAGCACGAGG	/56-FAM/TCCACAGAT/Zen/GGCTGATCGCTTACAAC/3IABkFQ/
gpx-5	GCGTACACCCAGCAATACA	TCAGCTGGCTCTTGCAAATA	/56-FAM/CCCAAGGAT/Zen/TTACTCTCATTGCCTTCCC/3IABkFQ/
gpdh-1	TTAGGGTGACAACGGATTATGAG	CAGACGGATGATAGCGGATTT	/56-FAM/TGGAGCTTT/Zen/GCGGAGCACTAAAGA/3IABkFQ/
cyp-37b1	GGCTGAAGCCTTTATGGTACT	GGAACTCTTCCCATTTCTCCTT	/56-FAM/CCCGGCCAT/Zen/GAGTATGACAGACAT/3IABkFQ/
f44e5.5	GCAGCTCGTAATCCAGAGAAT	CTCCAAATGGCCAATGCTTAC	/56-FAM/TGGACGACG/Zen/TTTCGATGAATCAACAGT/3IABkFQ/
lipl-5	CTCTGCGACAACGTCCTATT	CATGAGTAGCGTAGACTGGAAC	/56-FAM/AGTGACCAA/Zen/TGGAACCAGACACGT/3IABkFQ/
ncx-8	CCTGGGAATCTACGCAGTTTAT	TCACTTTCCAGCTGCTTCTT	/56-FAM/CCGTACTCG/Zen/GCGGAAGAACTTGAAA/3IABkFQ/
cyp13a6	GAAGTAGCTGTACAGGCAAGAA	GAACTCGGGCATCAAGGAATA	/56-FAM/AAAGGAGTT/Zen/GAGCCAGGAGAACCT/3IABkFQ/

* The expression level of each gene was normalized comparing to the expression of the *pmp3* (Ce02485188_m1) and *tba1* (Ce02412618_gH) genes from *Life Technologies* (TaqMan Gene Expression Assays), as housekeeping gene controls.

Table S2. Differentially expressed genes after treatment with resveratrol (200 µM) *vs* DMSO-control worms (ANOVA $p<0.05$; $-2 \leq FC \geq 2$). Results obtained in Transcriptomic Array Console (TAC, Affymetrix).

Transcript Cluster ID	FC (RSV	ANOVA p-value	Gene Symbol	Description
18484714	-13.27	0.0007	F49E12.10	Protein F49E12.10
18598738	-4.55	0.0025	lys-2	Protein LYS-2
18632007	-4.38	0.0045	pgp-6	Protein PGP-6
18484756	-4.16	0.0054	F49E12.9	Protein F49E12.9
18453349	-3.90	0.0010	acdh-1	Protein ACDH-1
18570161	-3.67	0.0068	CELE_F44G3.2	Protein F44G3.2
18597749	-3.64	0.0035	CELE_W03F9.4	Protein W03F9.4
18564293	-3.58	0.0051	C33G8.2	Protein C33G8.2
18615561	-3.56	0.0333	col-169	Protein COL-169
18567605	-3.21	0.0162	CELE_F20G2.5	Protein F20G2.5
18520934	-3.21	0.0249	C06E8.5	Protein C06E8.5
18609505	-3.06	0.0017	abu-10	Protein ABU-10
18480145	-3.05	0.0143	C30G12.2	Protein C30G12.2
18528133	-3.03	0.0161	T24C4.4	Protein T24C4.4
18484287	-3.00	0.0367	F44E5.5	Protein F44E5.5; Protein F44E5.4
18589632	-3.00	0.0142	folt-2	Protein FOLT-2
18581627	-2.99	0.0231	abu-1	Protein ABU-1
18615564	-2.98	0.0096	col-170	Protein COL-170
18554228	-2.97	0.0038	CELE_K08D8.4	Protein K08D8.4
18614569	-2.94	0.0121	abu-9	Protein PQN-57
18597331	-2.92	0.0404	hsp-16.1	Protein HSP-16.1
18582396	-2.91	0.0301	abu-8	Protein ABU-8
18574052	-2.90	0.0276	hacd-1	Protein HACD-1
18587679	-2.90	0.0401	CELE_F19B2.5	Protein F19B2.5
18523094	-2.90	0.0205	col-91	Protein COL-91
18568946	-2.87	0.0479	F32D8.7	Protein F32D8.7
18473813	-2.82	0.0043	CELE_W01A8.6	Protein W01A8.6
18575832	-2.77	0.0438	col-157	Protein COL-157
18601944	-2.74	0.0231	ZK6.11	Protein ZK6.11
18615544	-2.73	0.0100	col-167	Protein COL-167
18548494	-2.68	0.0349	swt-3	Protein SWT-3
18550679	-2.67	0.0229	D2096.6	Protein D2096.6
18571247	-2.66	0.0397	F56A4.3	Protein F56A4.3
18520577	-2.64	0.0161	tsp-2	Protein TSP-2
18589884	-2.58	0.0120	F41E6.11	Protein F41E6.11
18526132	-2.55	0.0111	ugt-62	Protein UGT-62
18552629	-2.54	0.0134	grl-4	Protein GRL-4
18498866	-2.53	0.0154	gst-14	Protein GST-14
18574047	-2.48	0.0438	pqn-54	Protein PQN-54
18575197	-2.47	0.0295	col-146	Protein COL-146
18633021	-2.47	0.0230	Y102A11A.7	Protein Y102A11A.7
18617379	-2.46	0.0122	Y70D2A.1	Protein Y70D2A.1
18576993	-2.45	0.0296	hsp-16.11	Protein HSP-16.11
18466529	-2.45	0.0333	phat-2	Protein PHAT-2
18581646	-2.45	0.0211	col-149	Protein COL-149
18502392	-2.45	0.0135	abu-11	Protein ABU-11
18513698	-2.44	0.0280	tyr-2	Protein TYR-2
18556277	-2.40	0.0046	T14A8.2	Protein T14A8.2
18533906	-2.40	0.0285	C18H7.11	Protein C18H7.11
18621038	-2.39	0.0137	C34E7.4	Protein C34E7.4

18581691	-2.39	0.0408	col-150	Protein COL-150
18603456	-2.38	0.0063	clc-1	Protein CLC-1
18516242	-2.36	0.0491	col-88	Protein COL-88
18525560	-2.35	0.0124	K04C2.5	Protein K04C2.5
18557521	-2.35	0.0406	col-120	Protein COL-120
18576356	-2.33	0.0180	T20D4.4	Protein T20D4.4
18492312	-2.33	0.0447	mlt-7	Protein MLT-7
18614621	-2.30	0.0369	R11.2	Protein R11.2
18455073	-2.29	0.0064	sra-17	Protein SRA-17
18595609	-2.27	0.0126	CELE_T06E4.8	Protein T06E4.8
18593334	-2.27	0.0098	cpt-4	Protein CPT-4
18590657	-2.26	0.0361	F48G7.8	Protein F48G7.8
18589217	-2.25	0.0082	F35B12.4	Protein F35B12.4 // piit-1
18611996	-2.24	0.0154	nas-38	Protein NAS-38
18626291	-2.23	0.0247	mxl-3	Protein MXL-3
18596111	-2.23	0.0141	pmp-5	Protein PMP-5
18597632	-2.22	0.0465	CELE_W02F12.2	Protein W02F12.2
18582416	-2.22	0.0192	abu-6	Protein ABU-6
18572639	-2.20	0.0041	K06H6.1	Protein K06H6.1
18567815	-2.19	0.0020	cth-1	Protein CTH-1
18543282	-2.18	0.0204	abu-5	Protein ABU-5
18528112	-2.17	0.0392	toh-1	Protein TOH-1
18482914	-2.17	0.0148	F23F1.2	Protein F23F1.2
18586880	-2.15	0.0210	fat-7	Protein FAT-7
18520595	-2.15	0.0377	tsp-1	Protein TSP-1
18603195	-2.14	0.0147	C06G1.1	Protein C06G1.1
18581856	-2.14	0.0302	col-145	Protein COL-145
18521602	-2.13	0.0023	CELE_C23G10.6	Protein C23G10.6
18622471	-2.12	0.0367	gcsh-1	Protein GCSH-1
18590783	-2.11	0.0261	oac-32	Protein OAC-32
18603588	-2.10	0.0186	C11E4.1	Protein C11E4.1
18465325	-2.09	0.0090	col-62	Protein COL-62
18552963	-2.09	0.0306	col-133	Protein COL-133
18454813	-2.09	0.0187	col-58	Protein COL-58
18547290	-2.08	0.0424	ZK593.3	Protein ZK593.3
18494406	-2.08	0.0304	acdh-2	Protein ACDH-2
18568239	-2.08	0.0451	asns-1	Protein ASNS-1
18505735	-2.08	0.0482	col-71	Protein COL-71
18589579	-2.08	0.0436	F36H9.5	Protein F36H9.5
18574697	-2.07	0.0020	ugt-53	Protein UGT-53
18451679	-2.07	0.0224	C15C6.1	Protein C15C6.1
18601975	-2.06	0.0261	lron-2	Protein LRON-2
18571942	-2.06	0.0425	unc-23	Protein UNC-23
18536789	-2.03	0.0052	lys-10	Protein LYS-10
18556853	-2.03	0.0282	pqn-74	Protein PQN-74
18525231	-2.03	0.0117	H10E21.4	Protein H10E21.4
18611252	-2.01	0.0493	aagr-4	Protein AAGR-4
18503706	-2.01	0.0289	T23F4.3	Protein T23F4.3
18452642	2.03	0.0122	fipr-22	Protein FIPR-22
18547230	2.04	0.0102	CELE_ZK550.2	Protein ZK550.2
18536036	2.04	0.0080	tpra-1	Protein TPRA-1
18629679	2.09	0.0058	M60.7	Protein M60.7
18494751	2.10	0.0131	tre-5	Protein TRE-5
18597214	2.11	0.0181	CELE_T26H5.8	Protein T26H5.8
18624593	2.16	0.0111	F25E2.3	Protein F25E2.3

18537273	2.16	0.0302	thn-2	Protein THN-2
18501194	2.22	0.0327	K09F6.5	Protein K09F6.5
18568638	2.27	0.0294	cyp-37B1	Protein CYP-37B1
18561523	2.64	0.0010	nlp-29	Protein NLP-29
18589254	2.91	0.0182	CELE_F35E12.5	Protein F35E12.5 //irg-5
18595142	2.92	0.0000	CELE_T03E6.8	Protein T03E6.8
18558751	3.01	0.0064	CELE_Y51H4A.5	Protein Y51H4A.5
18623122	3.16	0.0050	F09C8.1	Protein F09C8.1
18567774	3.33	0.0010	CELE_F21F8.4	Protein F21F8.4
18555883	3.96	0.0020	CELE_T07G12.5	Protein T07G12.5

Table S3. Main GO processes and KEGG signalling pathways affected by resveratrol (200 µM) treatment. Analysis obtained from EGAN UCSF software.

Gene Ontology Process	Process Name	Enrichment	KEGG	Pathway Name	Enrichment
GO:0006950	response to stress	5.45E-11	100	Steroid biosynthesis	0.0120
GO:0030968	endoplasmic reticulum unfolded protein response	1.12E-09	590	Arachidonic acid metabolism	0.0700
GO:0034620	cellular response to unfolded protein	2.66E-09	62	Fatty acid elongation in mitochondria	0.0756
GO:0006986	response to unfolded protein	3.49E-09	1040	Biosynthesis of unsaturated fatty acids	0.0922
GO:0034976	response to endoplasmic reticulum stress	3.49E-09	910	Nitrogen metabolism	0.1193
GO:0035967	cellular response to topologically incorrect protein	3.97E-09	500	Starch and sucrose metabolism	0.1456
GO:0006952	defense response	3.97E-09	650	Butanoate metabolism	0.1610
GO:0035966	response to topologically incorrect protein	1.31E-08	4141	Protein processing in endoplasmic reticulum	0.1655
GO:0050896	response to stimulus	2.60E-07	250	Alanine, aspartate and glutamate	0.1761
GO:0071310	cellular response to organic substance	8.33E-07	510	N-Glycan biosynthesis	0.1810
GO:0045087	innate immune response	1.97E-06	330	Arginine and proline metabolism	0.1958
GO:0006955	immune response	2.15E-06	310	Lysine degradation	0.1958
GO:0070887	cellular response to chemical stimulus	2.20E-06	480	Glutathione metabolism	0.2055
GO:0002376	immune system process	2.24E-06	380	Tryptophan metabolism	0.2103
GO:0010033	response to organic substance	4.25E-06	280	Valine, leucine and isoleucine degradation	0.2431
GO:0009617	response to bacterium	1.73E-05	71	Fatty acid metabolism	0.2876
GO:0042742	defense response to bacterium	1.73E-05	4010	MAPK signaling pathway	0.3172
GO:0033554	cellular response to stress	1.22E-04	1100	Metabolic pathways	0.5688
GO:0006633	fatty acid biosynthetic process	0.0587			
GO:0072330	monocarboxylic acid biosynthetic process	0.0701			
GO:1901136	carbohydrate derivative catabolic process	0.0812			
GO:0046394	carboxylic acid biosynthetic process	0.0977			

Table S4. Differentially expressed genes after apigenin (100 µM) treatment *vs* DMSO-control worms (ANOVA *p*<0.05; -1.5 ≤ FC ≥ 1.5). Results obtained in Transcriptomic array console (TAC, Affymetrix).

Transcript Cluster ID	FC (APIG vs. CNT)	ANOVA p-value (APIG vs. CNT)	Gene Symbol	Description
18548347	-3.12	0.004376	lact-6	Protein LACT-6
18497698	-2	0.035086	F16G10.11	Protein F16G10.11
18542887	-1.97	0.006494	CELE_W02A2.9	Protein W02A2.9
18464574	-1.97	0.011399	B0205.13	Protein B0205.13
18582764	-1.94	0.019364	cyp-35C1	Protein CYP-35C1
18560391	-1.91	0.025953	pqn-90	Protein PQN-90
18592542	-1.85	0.010530	CELE_K02H11.9	Protein K02H11.9
18599548	-1.84	0.000679	CELE_Y43F8B.14	Protein Y43F8B.14
18487591	-1.79	0.018389	CELE_T01B7.13	Protein T01B7.13
18564936	-1.77	0.003707	pgp-9	Protein PGP-9
18490903	-1.75	0.041863	Y51H7C.1	Protein Y51H7C.1
18573619	-1.74	0.021973	M03F8.1	Protein M03F8.1
18553047	-1.7	0.012934	CELE_F53B2.8	Protein F53B2.8
18582377	-1.69	0.019432	ugt-51	Protein UGT-51
18583320	-1.67	0.009720	ncx-8	Protein NCX-8
18616126	-1.67	0.010287	T22B7.3	Protein T22B7.3
18503466	-1.66	0.013107	fbxb-37	Protein FBXB-37
18498855	-1.65	0.007166	CELE_F36H5.9	Protein F36H5.9
18568638	-1.65	0.012400	cyp-37B1	Protein CYP-37B1
18523225	-1.64	0.014591	pqn-29	Protein PQN-29
18592847	-1.63	0.019143	K07B1.8	Protein K07B1.8
18565064	-1.62	0.022887	irg-2	Protein IRG-2
18625316	-1.61	0.016830	F38B2.6	Protein F38B2.6
18628994	-1.61	0.025156	cyp-14A2	Protein CYP-14A2
18457037	-1.6	0.003582	F56H6.1	Protein F56H6.1
18567575	-1.59	0.020037	srbc-27	Protein SRBC-27
18584695	-1.58	0.029665	dmd-11	Protein DMD-11
18594198	-1.56	0.000312	cyp-33C8	Protein CYP-33C8
18609993	-1.55	0.001214	F40E10.5	Protein F40E10.5
18561846	-1.55	0.013581	B0554.4	Protein B0554.4
18461357	-1.53	0.031891	CELE_Y18D10A.4	Protein Y18D10A.4
18495412	-1.53	0.048672	gba-2	Protein GBA-2
18550503	-1.51	0.040749	CELE_C53D6.6	Protein C53D6.6
18545035	-1.51	0.040928	Y54G2A.36	Protein Y54G2A.36
18607346	1.5	0.006976	pcyt-1	Protein PCYT-1
18574818	1.5	0.043886	T04C12.7	Protein T04C12.7
18505097	1.53	0.001050	clec-4	Protein CLEC-4
18626238	1.53	0.004780	CELE_F46F2.3	Protein F46F2.3
18600676	1.54	0.001062	CELE_Y69H2.1	Protein Y69H2.1
18607658	1.54	0.031808	scav-4	Protein SCAV-4
18496370	1.55	0.025311	D1022.3	Protein D1022.3
18558646	1.55	0.029226	Y45F10D.2	Protein Y45F10D.2
18626595	1.55	0.036069	F47B7.7	Protein F47B7.7
18569779	1.56	0.010778	CELE_F41B5.12	Protein F41B5.12
18559956	1.56	0.035711	CELE_Y69A2AR.27	Protein Y69A2AR.27
18575442	1.57	0.006905	CELE_T08G3.7	Protein T08G3.7
18574261	1.57	0.015710	pqn-60	Protein PQN-60
18542406	1.57	0.038379	T22B11.4	Protein T22B11.4

18476129	1.58	0.003490	Y51F10.7	Protein Y51F10.7
18561999	1.59	0.006068	CELE_C01G10.4	Protein C01G10.4
18623200	1.62	0.014630	F09F9.2	Protein F09F9.2
18614294	1.64	0.007574	scav-5	Protein SCAV-5
18459624	1.65	0.003595	T21G5.6	Protein T21G5.6
18477914	1.66	0.035780	B0228.1	Protein B0228.1
18538726	1.66	0.044014	noah-2	Protein NOAH-2
18627064	1.67	0.010550	wrt-2	Protein WRT-2
18580352	1.69	0.001353	glb-33	Protein GLB-33
18583384	1.69	0.011406	C13G3.1	Protein C13G3.1
18544174	1.7	0.035356	col-108	Protein COL-108
18490177	1.71	0.034250	nspe-7	Protein NSPE-7
18561500	1.72	0.002612	B0024.4	Protein B0024.4
18603602	1.73	0.001004	C11E4.2	Protein C11E4.2
18588166	1.73	0.006119	F22F7.8	Protein F22F7.8
18581342	1.73	0.043085	lipl-5	Protein LIPL-5
18472317	1.74	0.012859	R11A5.3	Protein R11A5.3
18623467	1.86	0.031159	F13C5.5	Protein F13C5.5
18628595	1.97	0.008982	aqp-8	Protein AQP-8
18558503	2.13	0.029505	CELE_Y43D4A.2	Protein Y43D4A.2
18494494	2.25	0.023256	CELE_C17F4.7	Protein C17F4.7
18505045	2.4	0.019494	nspe-1	Protein NSPE-1
18503106	2.81	0.000542	cyp-13A6	Protein CYP-13A6

Table S5. Main GO processes and KEGG signalling pathways affected by apigenin (100 μM) treatment. Analysis obtained from EGAN UCSF software.

Gene Ontology Process	Process Name	Enrichment	KEGG	Pathway Name	Enrichment
GO:0009617	Response to bacterium	0.00066	4020	Calcium signaling pathway	0.00797
GO:0042742	Defense response to bacterium	0.00066	564	Glycerophospholipid metabolism	0.01025
GO:0098542	Defense response to other organism	0.00115	600	Sphingolipid metabolism	0.01931
GO:0051707	Response to other organism	0.00118	100	Steroid biosynthesis	0.01946
GO:0009607	Response to biotic stimulus	0.00118	980	Metabolism of xenobiotics by cytochrome	0.03246
GO:0043207	Response to external biotic stimulus	0.00118	4142	Lysosome	0.03717
GO:0006952	Defense response	0.00252	982	Drug metabolism - cytochrome P450	0.03891
GO:0050829	Defense response to Gram-negative bacterium	0.00657	310	Lysine degradation	0.04819
GO:0009605	Response to external stimulus	0.02484	480	Glutathione metabolism	0.05311
GO:0006950	Response to stress	0.03573	4320	Dorso-ventral axis formation	0.10245
GO:0051704	Multi-organism process	0.05546	71	Fatty acid metabolism	0.10398
GO:0050830	Defense response to Gram-positive bacterium	0.06091	590	Arachidonic acid metabolism	0.11123
GO:0018996	Molting cycle, collagen and cuticulin-based cuticle	0.07878	514	Other types of O-glycan biosynthesis	0.12854
GO:0042303	Molting cycle	0.07878	511	Other glycan degradation	0.12854
GO:0050896	Response to stimulus	0.16943	830	Retinol metabolism	0.13706
GO:0045132	Meiotic chromosome segregation	0.24482	53	Ascorbate and aldarate metabolism	0.14551
GO:0019915	Lipid storage	0.30035	860	Porphyrin and chlorophyll metabolism	0.15387
GO:0098813	Nuclear chromosome segregation	0.30199	40	Pentose and glucuronate interconversions	0.17846
GO:0051235	Maintenance of location	0.30460	1100	Metabolic pathways	0.20077
GO:0010876	Lipid localization	0.30601	4080	Neuroactive ligand-receptor interaction	0.21016
GO:0007059	Chromosome segregation	0.31791	500	Starch and sucrose metabolism	0.22554
GO:0048477	Oogenesis	0.34619	4070	Phosphatidylinositol signaling system	0.24063
			4012	ErbB signaling pathway	0.24807
			983	Drug metabolism - other enzymes	0.26994

Table S6. Differentially expressed genes after vanillic acid (100 µM) treatment *vs* DMSO-control worms (ANOVA $p<0.05$; $-1.5 \leq FC \geq 1.5$). Results obtained in Transcriptomic array console (TAC, Affymetrix).

Transcript Cluster ID	FC (VAN vs. CNT)	ANOVA p-value (VAN vs. CNT)	Gene Symbol	Description
18478405	2.31	0.020037	nspb-9	Protein NSPB-9
18565542	2.29	0.016357	C54D10.9	Protein C54D10.9
18484287	2.04	0.034070	F44E5.5; F44E5.4	Protein F44E5.5; Protein F44E5.4
18599811	1.98	0.011147	hsp-16.2	Protein HSP-16.2
18478418	1.94	0.046852	nspb-7	Protein NSPB-7
18516585	1.85	0.012236	fbxa-61	Protein FBXA-61
18603602	1.84	0.002737	C11E4.2	Protein C11E4.2
18601841	1.8	0.039633	CELE_ZK262.19	Protein ZK262.19
18602807	1.73	0.048613	nspc-7	Protein NSPC-7
18516830	1.71	0.009942	fmo-3	Protein FMO-3
18456088	1.7	0.012980	gpdh-1	Protein GPDH-1
18483501	1.7	0.025977	nspb-11	Protein NSPB-11
18583050	1.7	0.022649	str-130	Protein STR-130
18563830	1.69	0.027037	C25F9.9	Protein C25F9.9
18493317	1.67	0.021547	nspb-10	Protein NSPB-10
18539599	1.65	0.029163	nspb-6	Protein NSPB-6
18589217	1.63	0.041958	F35B12.4	Protein F35B12.4
18629289	1.62	0.044716	K10C2.14	Protein K10C2.14
18573963	1.61	0.043645	CELE_R08A2.7	Protein R08A2.7
18614327	1.59	0.032859	R07B1.9	Protein R07B1.9
18622500	1.59	0.047424	D1053.3	Protein D1053.3
18532772	1.56	0.046983	CELE_C01B10.44	Protein C01B10.44
18550546	1.55	0.001434	C54E4.4	Protein C54E4.4
18535549	1.54	0.021219	C49A9.9	Protein C49A9.9
18580352	1.54	0.022197	glb-33	Protein GLB-33
18610156	1.54	0.027160	cnc-8	Protein CNC-8
18544174	1.53	0.017068	col-108	Protein COL-108
18564629	1.53	0.039761	sru-27	Protein SRU-27
18589254	1.53	0.039303	CELE_F35E12.5	Protein F35E12.5
18485054	1.52	0.002675	aat-7	Protein AAT-7
18582648	1.52	0.007568	ugt-63	Protein UGT-63
18513858	1.51	0.017342	K10D2.7	Protein K10D2.7
18576999	1.51	0.010980	hsp-16.48; hsp-16.49	Protein HSP-16.48; Protein HSP-16.49
18597334	1.51	0.010980	hsp-16.48; hsp-16.49	Protein HSP-16.48; Protein HSP-16.49
18550503	-1.5	0.018547	CELE_C53D6.6	Protein C53D6.6
18592291	-1.5	0.048792	srbc-40	Protein SRBC-40
18578524	-1.52	0.026553	CELE_Y37H2C.4	Protein Y37H2C.4
18606692	-1.52	0.005479	C54G7.1	Protein C54G7.1
18602419	-1.53	0.003016	B0416.2	Protein B0416.2

18631683	-1.53	0.020527	dop-3	Protein DOP-3
18570288	-1.54	0.023570	sru-39	Protein SRU-39
18564416	-1.55	0.043030	C35A11.3	Protein C35A11.3
18592611	-1.55	0.023098	srh-118	Protein SRH-118
18494801	-1.57	0.020272	CELE_C24H12.6	Protein C24H12.6
18576386	-1.58	0.038045	T20D4.8	Protein T20D4.8
18578207	-1.6	0.030162	CELE_Y17D7C.1	Protein Y17D7C.1
18568638	-1.63	0.017153	cyp-37B1	Protein CYP-37B1
18590701	-1.63	0.014059	CELE_F49H6.12	Protein F49H6.12
18563387	-1.67	0.035391	C17B7.3	Protein C17B7.3
18541006	-1.72	0.047797	M70.2	Protein M70.2
18522338	-1.75	0.026676	arl-6	Protein ARL-6
18630450	-1.78	0.031722	pqn-57; abu-9	Protein PQN-57; Protein ABU-9
18458379	-1.98	0.049408	CELE_M01G12.5	Protein M01G12.5
18562596	-1.98	0.025431	fbxa-157	Protein FBXA-157

Table S7. Main GO processes and KEGG signalling pathways affected by vanillic acid (100 μM) treatment. Analysis obtained from EGAN UCSF software.

Gene Ontology Process	Process Name	Enrichment	KEGG	Pathway Name	Enrichment
GO:0050830	defense response to Gram-positive bacterium	0.03699	4122	Sulfur relay system	0.01248
GO:0030968	endoplasmic reticulum unfolded protein response	0.05985	590	Arachidonic acid metabolism	0.01360
GO:0034620	cellular response to unfolded protein	0.06628	982	Drug metabolism - cytochrome P450	0.03589
GO:0006986	response to unfolded protein	0.06842	480	Glutathione metabolism	0.04248
GO:0034976	response to endoplasmic reticulum stress	0.06842	564	Glycerophospholipid metabolism	0.05120
GO:0035967	cellular response to topologically incorrect protein	0.06948			
GO:0035966	response to topologically incorrect protein	0.08008			
GO:0009617	response to bacterium	0.09783			
GO:0042742	defense response to bacterium	0.09783			
GO:0098542	defense response to other organism	0.11728			
GO:0051707	response to other organism	0.11830			
GO:0009607	response to biotic stimulus	0.11830			
GO:0043207	response to external biotic stimulus	0.11830			
GO:0071310	cellular response to organic substance	0.13236			
GO:0070887	cellular response to chemical stimulus	0.14915			
GO:0010033	response to organic substance	0.16178			
GO:0006950	response to stress	0.17301			
GO:0033554	cellular response to stress	0.24618			
GO:0006952	defense response	0.29435			
GO:0009605	response to external stimulus	0.31772			
GO:0007165	signal transduction	0.34949			
GO:0042221	response to chemical	0.37623			
GO:0050896	response to stimulus	0.37748			
